# Promotion and Detection of Cell–Cell Interactions through a Bioorthogonal Approach

**DOI:** 10.1021/jacs.4c04317

**Published:** 2024-05-20

**Authors:** Evelyn Y. Xue, Alan Chun Kit Lee, Kwan T. Chow, Dennis K. P. Ng

**Affiliations:** † Department of Chemistry, The Chinese University of Hong Kong, Shatin, N.T., Hong Kong, China; ‡ School of Life Sciences, The Chinese University of Hong Kong, Shatin, N.T., Hong Kong, China; § Department of Applied Biology and Chemical Technology, The Hong Kong Polytechnic University, Kowloon, Hong Kong, China; ∥ Department of Biomedical Sciences, City University of Hong Kong, Kowloon, Hong Kong, China

## Abstract

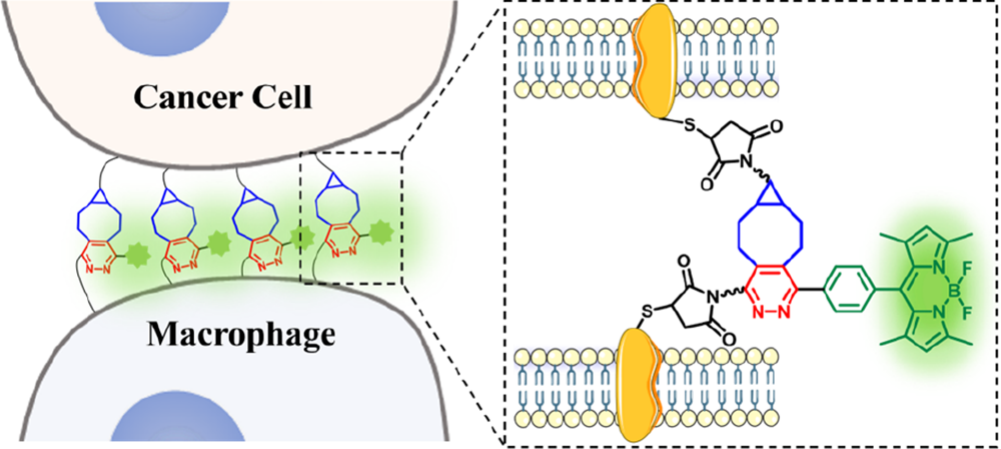

Manipulation of cell–cell interactions via cell surface modification is crucial in tissue engineering and cell-based therapy. To be able to monitor intercellular interactions, it can also provide useful information for understanding how the cells interact and communicate. We report herein a facile bioorthogonal strategy to promote and monitor cell–cell interactions. It involves the use of a maleimide-appended tetrazine-caged boron dipyrromethene (BODIPY)-based fluorescent probe and a maleimide-substituted bicyclo[6.1.0]non-4-yne (BCN) to modify the membrane of macrophage (RAW 264.7) and cancer (HT29, HeLa, and A431) cells, respectively, via maleimide–thiol conjugation. After modification, the two kinds of cells interact strongly through inverse electron-demand Diels–Alder reaction of the surface tetrazine and BCN moieties. The coupling also disrupts the tetrazine quenching unit, restoring the fluorescence emission of the BODIPY core on the cell–cell interface, and promotes phagocytosis. Hence, this approach can promote and facilitate the detection of intercellular interactions, rendering it potentially useful for macrophage-based immunotherapy.

## Introduction

Physical cell–cell interactions are fundamentally important in regulating diverse biological processes in multicellular organisms, such as tissue morphogenesis, neurotransmission, and immune response.^1,2^ The breakdown of cell communications and interactions may cause developmental defects, cancers, and neurological and immunological disorders. Therefore, technologies that enable manipulation and monitoring of direct cell–cell contact are indispensable for studying and controlling the cell behavior and advancing cell-based therapy.^3,4^ Accordingly, intense effort has been devoted to developing strategies to modify cell surfaces in a specific manner for controlling the intercellular interactions, which could have a huge impact in tissue engineering, regenerative medicine, and cellular immunotherapy. The chimeric antigen receptor (CAR) introduced to patients’ own T cells is probably the most notable example.^5^ Human macrophages engineered with CARs have also been reported to promote their interactions with cancer cells, thereby enhancing their phagocytic activity against tumors.^6^ Apart from this genetic engineering approach, various biology and chemistry-based strategies have also been developed for introducing non-native receptors and ligands to cell surfaces so as to manipulate the cell–cell interactions.^4,7^

In biological systems, cells generally come into contact randomly during migration. This transient contact, however, may not initiate any communications between them and the formation of cell aggregates. In fact, intercellular interactions are typically induced by the adhesion proteins on the surface of each cell partners.^8^ To facilitate manipulation of the interactions, molecules of complementary components, such as avidin and biotin,^9^ complementary oligonucleotides,^10−12^ and host–guest species^13−15^ have been artificially incorporated to the membrane of the cells. Alternatively, bispecific antibodies have also been used to connect two different cells having the corresponding surface antigens.^16^ Although the supramolecular interactions of these components occur spontaneously and are relatively strong, they may not be able to maintain stable cell aggregates in the biological environment, especially in vivo, under which they can be easily degraded by enzymes, such as proteases, DNases, and RNases.

In recent years, bioorthogonal chemistry has emerged as a facile and versatile tool for modulation of cell–cell interactions.^17^ It involves the introduction of two complementary bioorthogonal functionalities to two different kinds of cells separately through metabolic glycoengineering, membrane insertion, or coupling with the cell surface amine groups. After the click reaction, the cells can be covalently connected. For example, the strain-promoted azide–alkyne cycloaddition (SPAAC) reaction of bicyclo[6.1.0]non-4-yne (BCN)-modified tumor cells and azide-modified T cells^18^ or CAR-T cells^19^ has been utilized to enhance the recognition, migration, and cytotoxicity of the T cells. As the formation of covalent bonds is irreversible, it could lead to more stable cell aggregates than those being held by noncovalent bonding interactions.^20^ Furthermore, the small bioorthogonal functionalities can be introduced to the cell surface with high density, which can yield strong and multivalent adhesion between the cells. Compared with the methodologies involving complementary biomolecules as mentioned above,^9−12,16^ this approach could reduce the cost of synthesis and storage of the coupling reagents.

While a number of methodologies have been established for manipulation of cell–cell interactions, detection of physically interacting cells remains challenging in complex tissues.^21^ As early as 2008, Feinberg et al. genetically fused two nonfluorescent complementary fragments of green fluorescent protein (GFP) to the interacting cell partners.^22^ When the cells came into close contact, the split proteins combined to emit the GFP fluorescence as a reporter for detection of intercellular interactions. This methodology, namely GFP reconstitution across synaptic partners (GRASP), can also image synaptic locations and help to map synaptic connectivity in complex nervous systems.^23,24^ Another technology named GFP-based Touching Nexus was also developed for tracking physical interactions between cells.^25^ It involves the transfer of genetically engineered GFP from the sender cells to the receiver cells when they touch, enabling detection of the physical contacts. In addition, a synthetic Notch receptor was also introduced for visualization and genetic manipulation of the neighboring cells in vivo.^26^ Recently, a number of proximity-dependent labeling strategies have also been developed and emerged as powerful tools for monitoring and recording intercellular interactions in diverse living systems.^27−33^ However, they usually involve complicated processes relying on genetic manipulation, the presence of specific surface glycans to introduce cell-tagging enzymes, and/or toxic reagents, such as H_2_O_2_. We report herein a straightforward, general, and versatile approach for promotion and detection of cell–cell interactions, using inverse electron-demand Diels–Alder (iEDDA) reaction for covalently connecting the cells and remarkably activating the fluorescence emission of the probe for visualization of the interactions.

## Results and Discussion

### Working Principle

**Figure 1:**
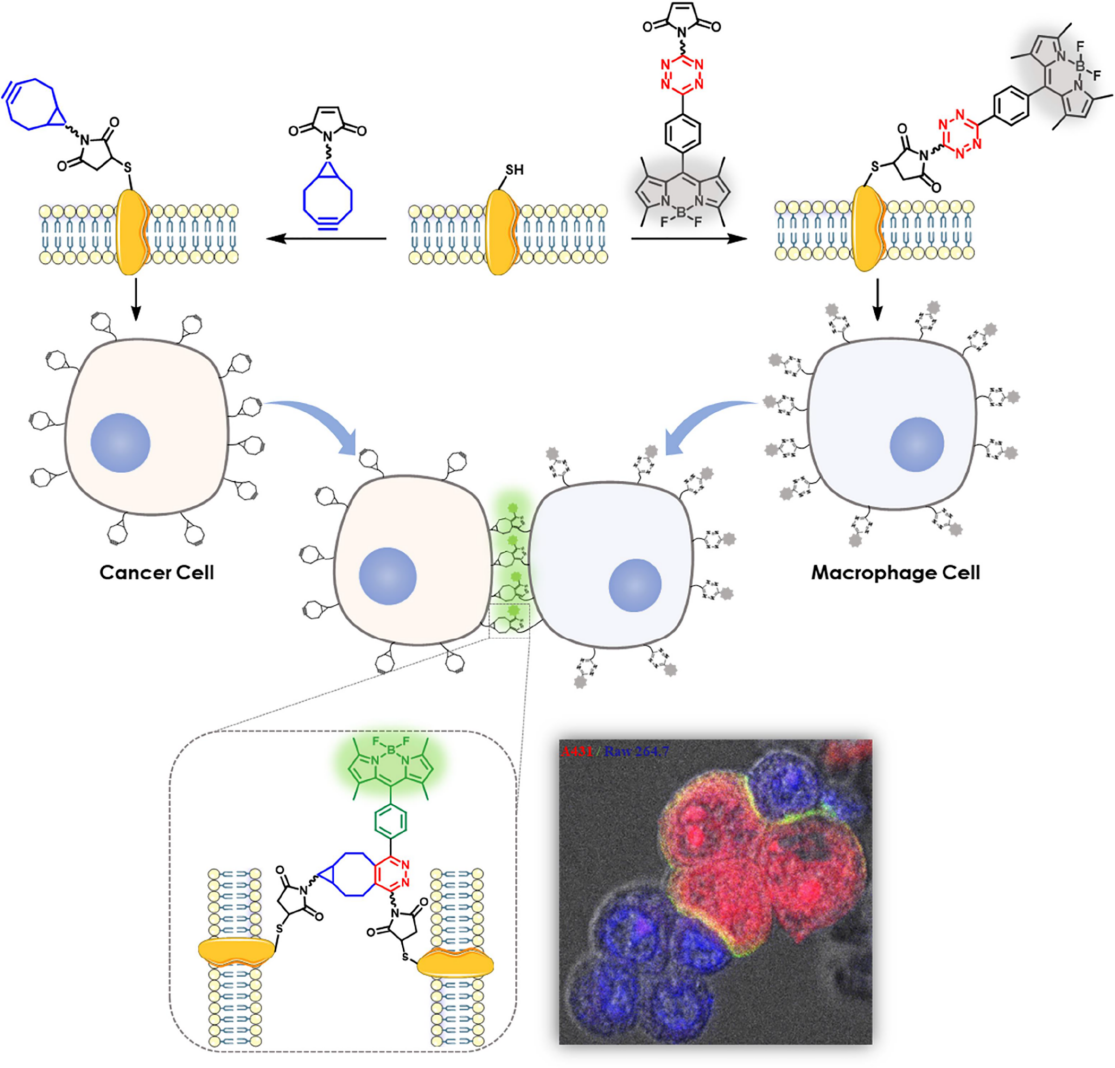
Working principle of the bioorthogonal strategy for promotion and detection of cell–cell interactions.

This bioorthogonal approach involves the use of a maleimide-modified tetrazine-caged boron dipyrromethene (BODIPY)-based fluorophore and a maleimide-substituted BCN to modify the plasma membrane surface of macrophage and cancer cells, respectively, through maleimide–thiol conjugation. The combination of immune and cancer cell types in this study enables demonstration of the potential application of this strategy for macrophage-based immunotherapy. After surface modification, these two kinds of cells can be connected readily through iEDDA reaction of the tetrazine and BCN moieties on the modified cell surfaces. In addition, the click reaction disrupts the tetrazine group, which can effectively quench the fluorescence of the BODIPY core by through-bond energy transfer,^34^ thereby restoring the fluorescence emission. This signal can light up the boundary of the direct cell–cell contact, revealing the existence of physical cell–cell interactions (Figure 1). Although bioorthogonal chemistry has been utilized to modulate intercellular interactions as mentioned above,^17^ and bioorthogonal activation of tetrazine-caged fluorophores has also been reported previously,^34,35^ the integration of these concepts for both promotion and detection of cell–cell interactions has not been reported so far. These dual functions can facilitate the studies to enhance our understanding of biological processes. By using macrophages, this strategy can also boost their phagocytic activity against cancer cells, making it potentially useful in cancer immunotherapy.

### Molecular Design and Synthesis

Given the high biocompatibility and extremely fast kinetics of iEDDA reactions,^36^ the 1,2,4,5-tetrazine and BCN moieties were selected as the complementary bioorthogonal functionalities. The former is also an effective quencher for various fluorophores.^34,35^ Both our research group and others have previously demonstrated that BODIPY-based fluorophores^34^ and photosensitizers^37,38^ can be largely quenched by this substituent, and their photoactivities can be effectively restored upon iEDDA reaction with *trans*-cyclooct-4-enes or BCNs to give the corresponding 1,4-dihydropyridazines or pyridazines. Hence, a tetrazine-caged BODIPY-based fluorophore was chosen as the activatable reporter, and a BCN was used as the activator. To immobilize these molecules on the cell surface, a maleimide group was introduced to couple with the cell surface thiol groups through maleimide–thiol conjugation. Owing to the high selectivity and fast kinetics in aqueous media as well as the robustness of the coupled product, the maleimide–thiol Michael addition reaction is a powerful and highly efficient conjugation method that has been widely used in various biological systems.^39,40^ A wide range of biomolecules, polymers, and nanoparticles have been immobilized on the cell surface using this click reaction.^41,42^ To enhance the flexibility for the click reaction, a hydrophilic peptide sequence was inserted between the two functionalities as a spacer.

**Scheme 1:**
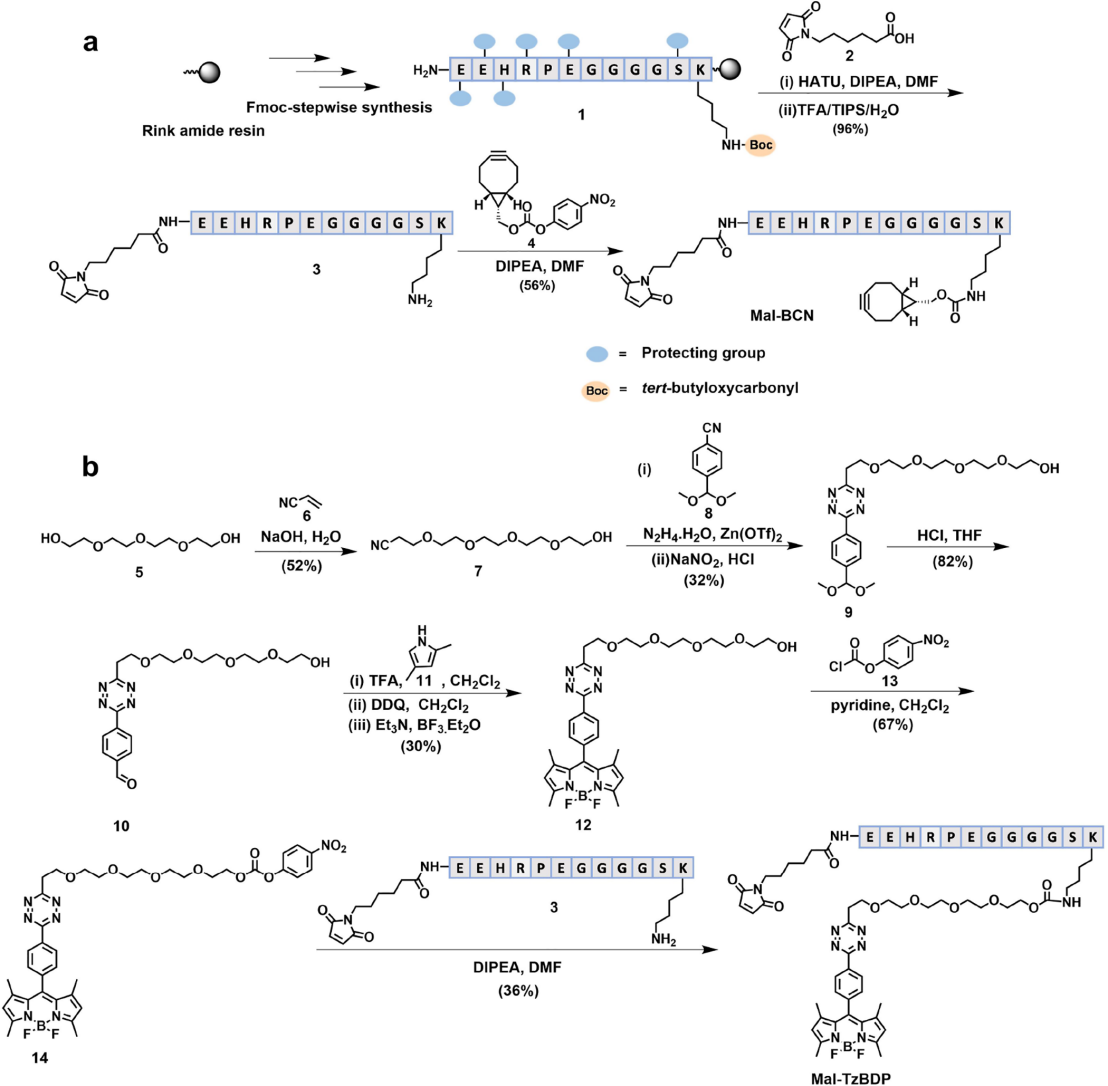
Synthetic Routes for (a) **Mal-BCN** and (b) **Mal-TzBDP**

Scheme 1a shows the synthetic route for the maleimide-substituted BCN (**Mal-BCN**). The peptide linker contained a terminal lysine (K) to facilitate the coupling with the BCN unit, a segment of oligoglycine and serine (GGGGS) that is commonly used as a spacer^43,44^ to further enhance the flexibility, and three anionic glutamate (E) to hinder the internalization of the resulting conjugate. To reduce the electrostatic repulsion arising from these anionic residues between the two segments connected to BCN and tetrazine moieties, respectively, which would affect the bioorthogonal coupling efficiency, two cationic arginine (R) and protonated histidine (H) residues were inserted between the glutamate residues. These amino acids were selected based on these considerations. We believe that the exact sequence might not be important as long as the lysine is placed at the C-terminal to ensure that the BCN moiety would not be too close to the cell surface that would hinder the bioorthogonal coupling. Resin **1**, which contained the overall sequence EEHRPEGGGGSK (with an additional proline (P) randomly added) was first prepared using a standard 9-fluorenylmethoxycarbonyl (Fmoc) solid-phase peptide synthesis (SPPS) protocol. The N-terminal of the resin was then coupled with 6-maleimidohexanoic acid (**2**) in the presence of 1-[bis(dimethylamino)methylene]-1H-1,2,3-triazolo[4,5-*b*]pyridinium 3-oxid hexafluorophosphate (HATU) and *N*,*N*-diisopropylethylamine (DIPEA) in *N*,*N*-dimethylformamide (DMF), followed by the detachment from the resin and removal of all the protecting groups upon treatment with a mixture of trifluoroacetic acid (TFA, 95%), triisopropylsilane (TIPS, 2.5%), and H_2_O (2.5%) to afford peptide **3.** It was then condensed with BCN **4**(45) in the presence of DIPEA to give the target conjugate **Mal-BCN**.

The maleimide-modified tetrazine-caged BODIPY (**Mal-TzBDP**) was synthesized according to Scheme 1b. Tetraethylene glycol (**5**) was first treated with acrylonitrile (**6**) in the presence of NaOH to give the nitrile-substituted analogue **7**. Treatment of this compound with NH_2_NH_2_·H_2_O and benzonitrile **8**(46) using Zn(OTf)_2_ (OTf = trifluoromethanesulfonate) as the catalyst, followed by oxidation with NaNO_2_ in an acidic condition led to the formation of the tetrazine derivative **9**. The acetal group of **9** was then removed upon treatment with HCl in tetrahydrofuran (THF) to give the tetrazine-substituted benzaldehyde **10**. This compound then underwent acid-promoted condensation with 2,4-dimethylpyrrole (**11**), followed by oxidation with 2,3-dichloro-5,6-dicyano-1,4-benzoquinone (DDQ) and complexation with BF_3_·Et_2_O to afford BODIPY **12**. Upon treatment with 4-nitrophenyl chloroformate (**13**) and pyridine, this compound was converted to **14**, which was then condensed with the maleimide-modified peptide **3** to afford **Mal-TzBDP**.

### Bioorthogonal Activation of Mal-TzBDP by BCNs

**Figure 2:**
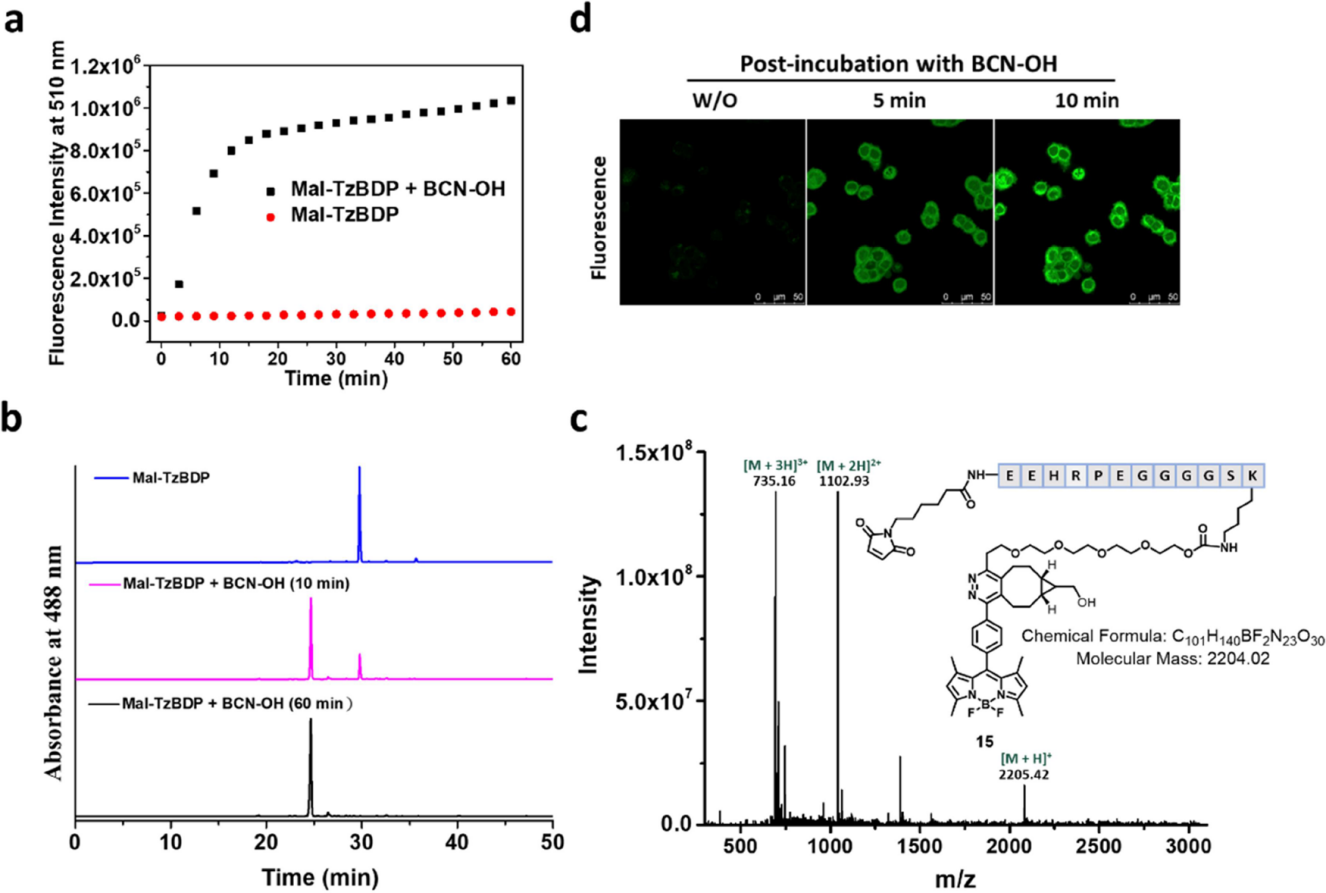
(a) Change in fluorescence intensity at 510 nm for **Mal-TzBDP** (2 μM) in the absence and presence of **BCN–OH** (10 μM) in PBS at pH 7.4 over a period of 60 min (λ_ex_ = 488 nm). (b) HPLC chromatograms of **Mal-TzBDP** (2 μM) and its reaction mixture with **BCN–OH** (10 μM) in PBS at pH 7.4 at 37 °C after mixing for 10 and 60 min. (c) ESI mass spectrum of the fraction with a retention time of 24.7 min and the molecular structure of **15**. (d) Confocal fluorescence images of HT29 cells after incubation with **Mal-TzBDP** (4 μM) for 30 min with or without postincubation with **BCN–OH** (10 μM) for 5 or 10 min.

The BCN-responsive property of **Mal-TzBDP** was first studied by fluorescence spectroscopy. This compound showed negligible fluorescence in phosphate-buffered saline (PBS) at pH 7.4, and the fluorescence intensity remained unchanged over a period of 60 min. In contrast, upon addition of 5 equiv of (1R,8S,9s)-bicyclo[6.1.0]non-4-yn-9-ylmethanol, labeled as **BCN–OH**, the fluorescence of **Mal-TzBDP** increased significantly over the same period of time (Figure S1 in the Supporting Information), which could be attributed to the removal of the tetrazine quencher upon bioorthogonal coupling.^34,37,38^ As shown in Figure 2a, the fluorescence intensity (at 510 nm) almost reached a plateau after about 15 min, showing that the click reaction proceeded quickly.

The mixture of **Mal-TzBDP** and **BCN–OH** (5 equiv) in PBS was further analyzed by liquid chromatography - mass spectrometry (LCMS) during the course of the reaction. As shown in Figure 2b, a new signal at a retention time of 24.7 min appeared after 10 min, while the signal for **Mal-TzBDP** (at 29.2 min) largely diminished. The latter virtually disappeared after 60 min, leaving the former as the only visible signal. Its electrospray ionization (ESI) mass spectrum showed the base peak at *m*/*z* 735 and two notable signals at *m*/*z* 1103 and 2205, which could be unambiguously assigned to the [M + 3H]^3+^, [M + 2H]^2+^, and [M + H]^+^ ions of the clicked product **15** (Figure 2c). These results confirmed the presence of iEDDA reaction between **Mal-TzBDP** and **BCN–OH**, and the reaction was completed within 60 min, which were fully consistent with the results obtained by using fluorescence spectroscopy. It is worth mentioning that maleimides are subject to ring-opening hydrolysis,^47^ but such a hydrolyzed product of **Mal-TzBDP** was not detected by LCMS. Kirchhof et al. reported that the rate of the hydrolysis increases with increasing the pH value and temperature. For a star-shaped PEGylated maleimide, the half-life was 929 min at pH 7.4 at 20 °C,^48^ which are roughly the conditions we used. The slow reaction could explain the absence of hydrolyzed product of **Mal-TzBDP** within 60 min, confirming that the maleimide group remains intact for cell-surface modification.

The study was then extended to the cellular level, using HT29 human colorectal adenocarcinoma cells as the first cell line. The cells were incubated with **Mal-TzBDP** (4 μM) for 30 min, followed by incubation with **BCN–OH** (10 μM) for different periods of time. As shown in Figure 2d, without the postincubation, the fluorescence of the cells was almost invisible. The cells, however, lit up significantly upon postincubation with **BCN–OH** for 5 min, and the fluorescence intensity increased when the incubation time was extended to 10 min. It is worth noting that fluorescence was present mainly in the cytoplasm, indicating that both **Mal-TzBDP** and **BCN–OH** were internalized into the cells. This observation showed that the bioorthogonal activation could also take place inside the cells. Despite the presence of a maleimide group, incubation of **Mal-TzBDP** did not lead to notable fluorescence on the cell membrane. It suggested that immobilization of the probe on the cell surface was minimal using this treatment, which could be attributed to the lack of thiol groups on the native cell membrane.

In an attempt to confine the activation on the cell surface, HT29 and HeLa human cervical carcinoma cells were first modified with **Mal-TzBDP**. It has been reported that tris(2-carboxyethyl)phosphine (TCEP) can partially reduce the disulfide bonds in membrane proteins to release free thiol groups, which can promote maleimide–thiol conjugation on the cell surface, and such surface modification would not adversely affect the cellular morphology, viability, proliferation, and metabolism when the concentration of TCEP is not higher than 1 mM.^41,42^ Therefore, we treated the two cell lines with TCEP (1 mM) for 30 min, followed by incubation with **Mal-TzBDP** (4 μM) for 30 min. The cells were then incubated in the culture medium with or without the presence of **BCN–OH** (10 μM) for 30 min. It was found that the intracellular fluorescence intensity increased remarkably upon further incubation with **BCN–OH**, and the fluorescence appeared both on the cell membrane and in the cytoplasm for both cell lines (Figure S2). The latter observation suggested that even with the TCEP treatment, part of **Mal-TzBDP** could still be internalized. Nevertheless, the notable fluorescence on the cell membrane suggested that this treatment could promote the anchoring of **Mal-TzBDP** on the cell surface likely due to the increased number of free thiol groups.

**Figure 3:**
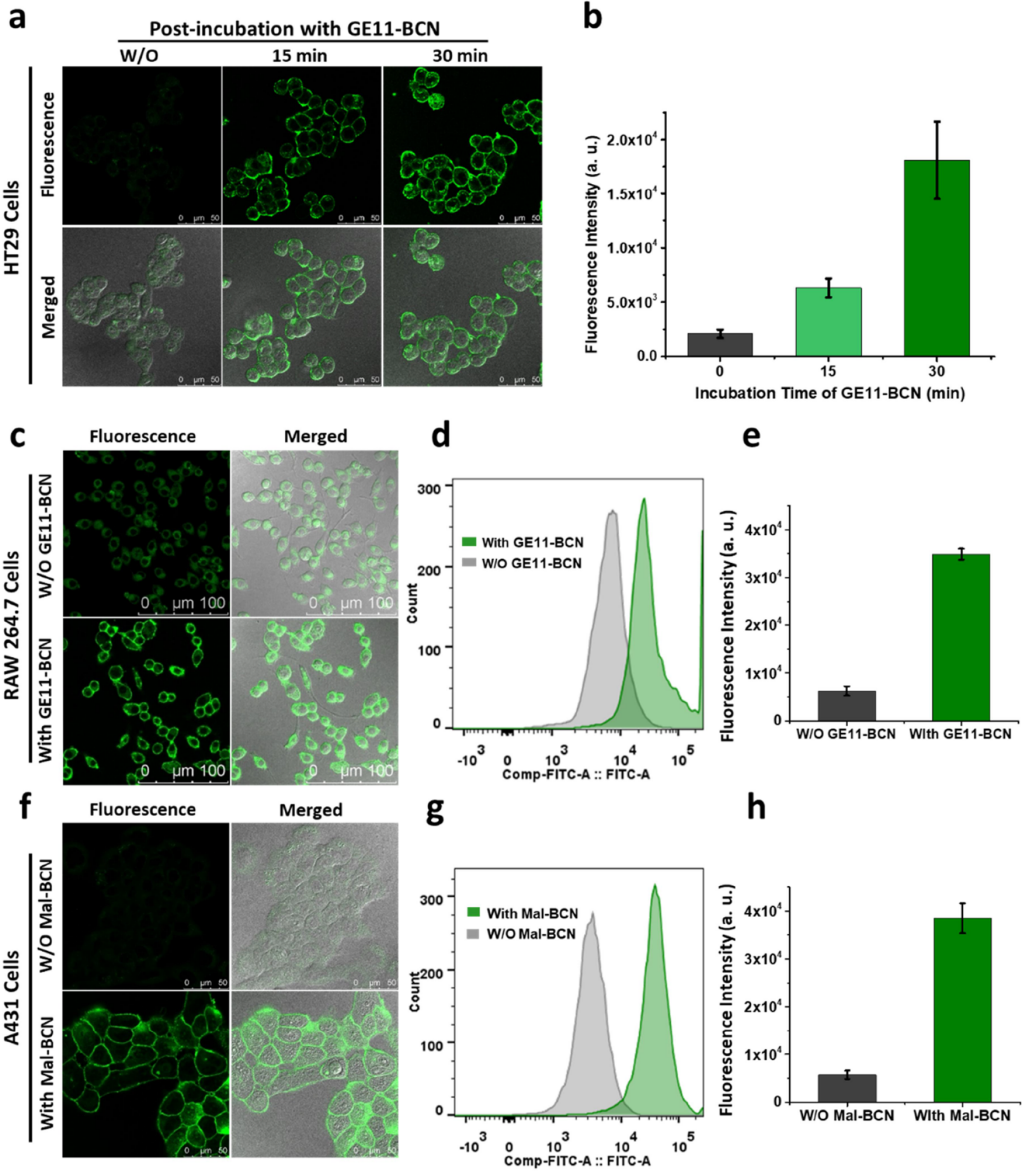
(a) Confocal images of HT29 cells after incubation with TCEP (1 mM) for 30 min and then with **Mal-TzBDP** (4 μM) for 30 min with or without further incubation with **GE11-BCN** (10 μM) for 15 or 30 min. (b) Corresponding quantified fluorescence intensities determined by flow cytometry. (c) Confocal images of RAW 264.7 cells after incubation with TCEP (1 mM) for 30 min and then with **Mal-TzBDP** (4 μM) for 30 min with or without further incubation with **GE11-BCN** (10 μM) for 30 min. (d) Corresponding histograms showing the fluorescence intensities determined by flow cytometry. (e) Corresponding quantified fluorescence intensities determined by flow cytometry. (f) Confocal images of A431 cells with or without treatment with TCEP (1 mM) and **Mal-BCN** (10 μM) each for 30 min, followed by incubation with **Mal-TzBDP** (4 μM) for 30 min. (g) Corresponding histograms showing the fluorescence intensities determined by flow cytometry. (h) Corresponding quantified fluorescence intensities determined by flow cytometry. For (b), (e), and (h), data are reported as the mean ± standard deviation (SD) of three independent experiments.

To ensure the activation on cell membrane, an attempt was then made to confine the activator on the cell surface. Instead of using **BCN–OH**, which has a small molecular structure that will promote the internalization, the GE11 peptide with the sequence YHWYGYTPQNVI was used to conjugate BCN, and the resulting conjugate, labeled as **GE11-BCN**, was used as the activator (see Scheme S1 for its structure and synthesis). According to our previous finding,^49,50^ GE11-conjugated dyes can stay on the cell surface when they are used for incubation for a short period of time (20–60 min). It is likely that the GE11 peptide, which is a well-documented targeting group of epidermal growth factor receptor (EGFR),^51^ binds specifically to this receptor overexpressed on the surface of EGFR-positive cancer cells, retaining the conjugates on the cell membrane shortly before they are internalized through receptor-mediated endocytosis. Hence, the **Mal-TzBDP**-modified HT29 and HeLa cells, both EGFR-positive,^50,52^ were incubated with **GE11-BCN** (10 μM) for 15 or 30 min. The fluorescence images and intensities were then measured using confocal microscopy and flow cytometry and compared with those without postincubation with **GE11-BCN**. As shown in Figure 3a for the results of the HT29 cells, while fluorescence could hardly be observed for the cells without post-treatment with **GE11-BCN**, a strong green fluorescence signal was observed on the cell membrane when the cells were post-treated with **GE11-BCN**. As expected, the intensity increased significantly with a longer incubation time (Figure 3b). Similar results were observed for the **Mal-TzBDP**-modified HeLa cells (Figure S3). Therefore, the use of membrane-bound **GE11-BCN** can confine the bioorthogonal activation on the cell membrane, leaving the internalized **Mal-TzBDP** intact.

A similar study was then extended to macrophages to show the generality of this approach and its potential application in cancer immunotherapy. Macrophages are innate immune cells that are responsible for host defense and homeostasis maintenance.^53^ They can initiate the clearance of aberrant cells and pathogens through a series of receptor-mediated molecular interactions to trigger phagocytosis. Macrophages recognize the “eat-me” signals from aberrant cells and tether to their surface to initiate a phagocytic synapse, which leads to the engulfment of the aberrant cells through the pro-phagocytic signaling pathways.^54^ However, it has been found that many cancer cells express high levels of antiphagocytic molecules, such as CD47, to allow them to evade phagocytosis initiated by macrophages.^55^ Therefore, by promoting the interactions between macrophage and cancer cells, the macrophage-mediated therapeutics could become more effective.^56,57^ To this end, we modified the RAW 264.7 murine macrophage cells with TCEP (1 mM) and **Mal-TzBDP** (4 μM) and then treated the modified cells with **GE11-BCN** (10 μM) for 30 min as described above. As shown in Figure 3c–e, postincubation with **GE11-BCN** resulted in significantly higher fluorescence intensity mainly on the cell surface than the control group, i.e., without the post-treatment with **GE11-BCN**, showing that the surface of RAW 264.7 cells could also be modified with **Mal-TzBDP**, which could be effectively activated by the surface-bound **GE11-BCN**.

To examine whether **Mal-BCN** can also be immobilized on the cell surface and used for activation, A431 human squamous carcinoma cells were arbitrary chosen to be sequentially treated with TCEP (1 mM) and **Mal-BCN** (10 μM) each for 30 min, followed by incubation with **Mal-TzBDP** (4 μM) for a further 30 min. The native or unmodified A431 cells were also studied for comparison. As revealed by confocal microscopy and flow cytometry, the cells modified with **Mal-BCN** showed a strong fluorescence signal, and the intensity was almost 8-fold higher than that of the native cells (Figure 3f–h). It is worth noting that the fluorescence appeared virtually exclusively on the cell membrane in this case. This observation suggested that **Mal-BCN** could not be internalized. Otherwise, it would activate the internalized **Mal-TzBDP** to emit fluorescence in the cytoplasm after these treatments. The different uptake behavior of **Mal-TzBDP** and **Mal-BCN** could be attributed to their different molecular structures. The internalization of **Mal-TzBDP** might be a result of its enhanced amphiphilic character arising from the hydrophobic BODIPY unit and the hydrophilic tetraethylene glycol chain and peptide sequence. It has been well documented that molecules with an amphiphilic structure can pass through the lipid bilayer of cell membrane more readily than the hydrophilic and hydrophobic counterparts.^58^ In contrast, the small hydrophobic BCN moiety in **Mal-BCN** could not alter the hydrophilic character of the peptide chain. Its high solubility in aqueous media hindered the internalization within a short period of time (30 min). These results showed that **Mal-BCN** can also be used for cell modification and the sequence of incubation of the two bioorthogonal components would not affect the activation.

To verify whether the membrane-bound **Mal-BCN** could be internalized through the turnover of the cell surface proteins, A431 cells were sequentially incubated with TCEP, **Mal-BCN**, and **Mal-TzBDP** as described above, followed by incubation in neat medium for a further 1, 4, and 8 h, respectively. It was found that the fluorescence remained predominantly on the cell membrane after postincubation for up to 4 h, but it was extended to the cytoplasm after prolonged incubation for 8 h (Figure S4). The results showed that the BCN bioorthogonal tag was anchored on the cell membrane for at least 4 h.

The role of TCEP was further demonstrated by comparing the cellular fluorescence intensities under different conditions. We first treated HT29 and RAW 264.7 cells sequentially with **Mal-TzBDP** (4 μM) and **Mal-BCN** (10 μM) each for 30 min, as well as A431 cells in reverse order. Without preincubation with TCEP, these treatments led to very weak fluorescence for all the three cell lines (Figure S5a). In contrast, upon preincubation with TCEP (1 mM) for 30 min, bright fluorescence could be observed in all the cases as shown in Figure 3. Figure S5b–d displayed a comparison of the quantified fluorescence intensities of these cells with or without the pretreatment with TCEP as determined by flow cytometry. It is clear that TCEP could largely increase the fluorescence intensity, confirming its role in promoting the generation of thiol groups on the cell surface.

Before applying this approach for studying intercellular interactions, the cytotoxicity of the thiol-generating agent TCEP and the two bioorthogonal components was examined using a MTT cell viability assay [MTT = 3-(4,5-dimethylthiazol-2-yl)-2,5-diphenyltetrazolium bromide]. As shown in Figure S6, TCEP was essentially noncytotoxic against HT29, A431, and RAW 264.7 cells up to 2 mM. Further surface modification of RAW 264.7 cells with **Mal-TzBDP** as well as A431 and HT29 cells with **Mal-BCN** did not cause a significant change in the cell viability up to 40 μM of the bioorthogonal tags (Figure S7). The results showed that the whole process of cell-surface modification, i.e., reduction by TCEP followed by maleimide–thiol conjugation, would not damage the cells and could be safely used for cell manipulation.

### Click-Induced Cell–Cell Interactions

**Figure 4:**
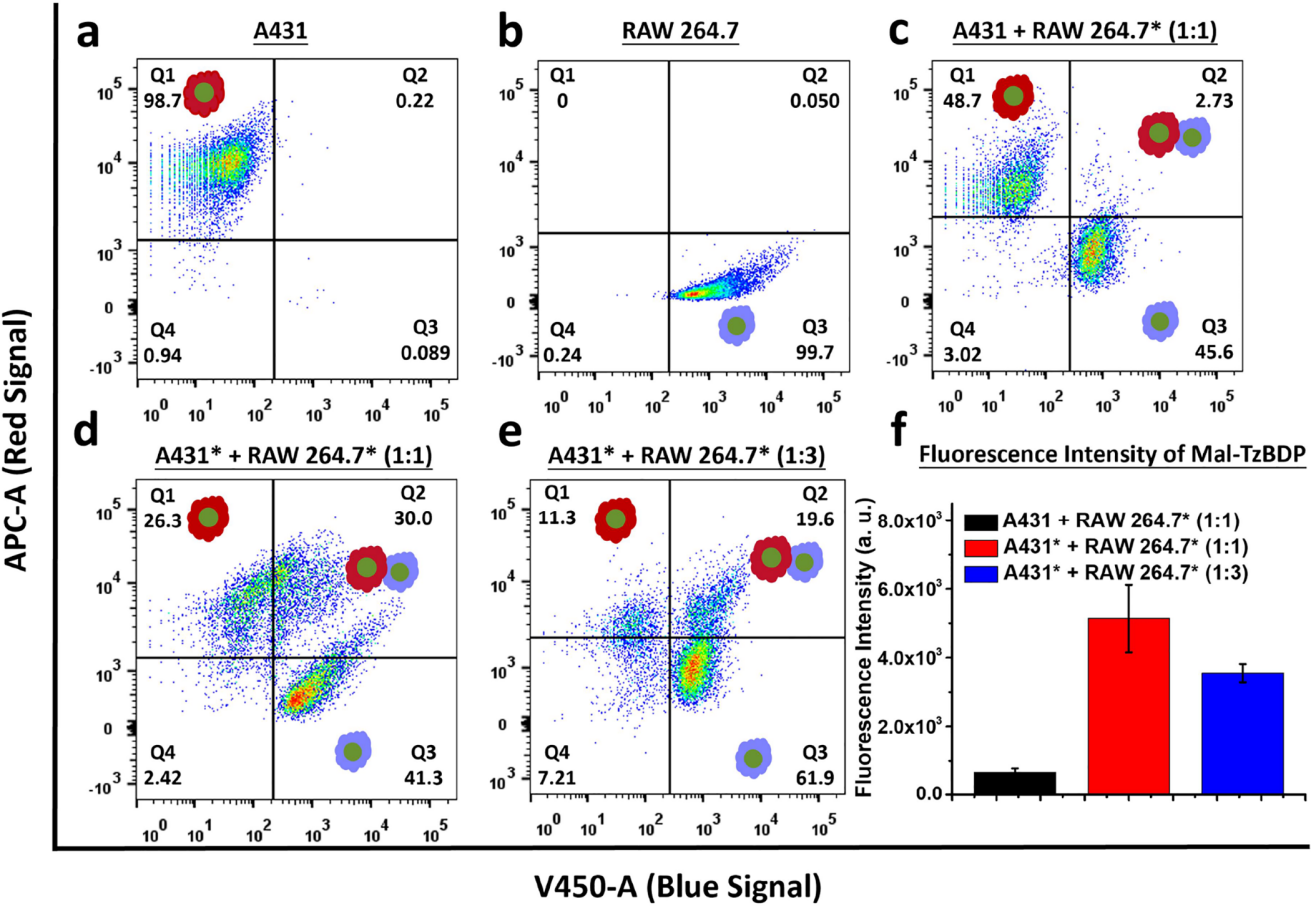
Flow cytometric analysis of different cell assemblies: (a) For native monocultured A431 cells. (b) For native monocultured RAW 264.7 cells. (c) For native A431 and RAW 264.7* cells cocultured in 1:1 ratio for 30 min. (d) For A431* (treated with 40 μM of **Mal-BCN**) and RAW 264.7* cells cocultured in 1:1 ratio for 30 min. (e) For A431* (treated with 40 μM of **Mal-BCN**) and RAW 264.7* cells cocultured in 1:3 ratio for 30 min. (f) Quantified fluorescence intensities of the activated **Mal-TzBDP** in the cell assemblies of (c) to (e). Data are reported as the mean ± SD of three independent experiments.

Based on these encouraging results, we further utilized this approach to induce and monitor cell–cell interactions. To facilitate the visualization, A431 and RAW 264.7 cells were stained with CellTracker Red CMTPX Dye and CellTrace Violet, respectively, before being modified on the surface. The stained A431 cells were sequentially treated with TCEP (1 mM) for 30 min and various concentrations of **Mal-BCN** (10, 20, or 40 μM) for 30 min to give **Mal-BCN**-modified A431 cells, labeled as A431* cells. Similarly, the stained RAW 264.7 cells were treated with TCEP (1 mM) and **Mal-TzBDP** (8 μM) each for 30 min to give **Mal-TzBDP**-modified RAW 264.7 cells, labeled as RAW 264.7* cells. The use of a higher concentration of **Mal-TzBDP** (2-fold) was aimed to increase the number of tetrazine tags on the cell surface that could promote the binding with cancer cells. Flow cytometry was first used to quantitatively analyze the connecting efficiency of the two kinds of cells in a large population (approximately 1 × 10^4^ cells). As shown in Figure 4a,b, the stained A431 and RAW 264.7 monocultured cells were mainly distributed in the upper left quadrant (Q1, with positive red fluorescence) and the lower right quadrant (Q3, with positive blue fluorescence) of the scatter plot, respectively. Figure 4c shows the scatter plot for a mixture of the native A431 and RAW 264.7* cells cocultured in 1:1 ratio for 30 min. No significant signal could be detected in the upper right quadrant (Q2, with positive red and positive blue fluorescence), showing that most of the cells remained separated. In contrast, for the mixture with A431* cells (treated with 40 μM of **Mal-BCN**), 30% of the cells appeared in the upper right quadrant (Figure 4d), which clearly indicated the occurrence of aggregates of the two cell types. The population in this quadrant increased gradually (from 2% to 25%) when a higher concentration of **Mal-BCN** (from 0 to 40 μM) was used for modification of A431 cells as shown in Figure S8a. In addition, we also studied a mixture of A431* (treated with 40 μM of **Mal-BCN**) and RAW 264.7* cells in 1:3 ratio (Figure 4e). Although the connecting efficiency as reflected by the cell population in Q2 decreased to 20%, the percentage of free A431* cells dropped to 11%. More importantly, the ratio of RAW 264.7* cell-bound and free A431 cells, i.e., the ratio of cell population in Q2 vs Q1, increased from 1.1 to 1.7 when the ratio of the two kinds of cells was changed from 1:1 to 1:3. Hence, a larger population of RAW 264.7* cells could bind to more A431* cells, which could enhance the macrophage-mediated therapeutic efficacy.

The fluorescence intensities of the activated **Mal-TzBDP** were also measured by flow cytometry under these conditions. As shown in Figure 4f, the fluorescence intensity for the 1:1 mixture of A431* and RAW 264.7* cells was ca. 1.5-fold higher than that for the 1:3 cell mixture, and the intensities for both of these conditions were much higher than that for the 1:1 cell mixture with native A431 cells. This trend was in good agreement with their relative connecting efficiency determined by the double-staining method mentioned above.

Similar study was also performed with HT29 cells. For a 1:1 cell mixture with RAW 264.7* cells, the population in Q2 was just 4% (Figure S8b). In contrast, by using HT29* cells instead, which were prepared by treating the native cells with TCEP (1 mM) and then with **Mal-BCN** (40 μM) each for 30 min, the population in Q2 increased significantly to 21% (Figure S8c). The fluorescence intensity of the activated **Mal-TzBDP** was also approximately 5-fold higher than that for the cell mixture with native HT29 cells (Figure S8d). Both results were consistent, which suggested the presence of click-induced assembly of HT29* and RAW 264.7* cells. The lower connecting efficiency for HT29* cells (21%) compared with that for A431* cells (30%) might be due to the fewer disulfide groups on the cell membrane of HT29 cells than that of A431 cells. All the above results demonstrated that this bioorthogonal approach can promote the interactions between RAW 264.7* cells and different cancer cells within a short period of time.

**Figure 5:**
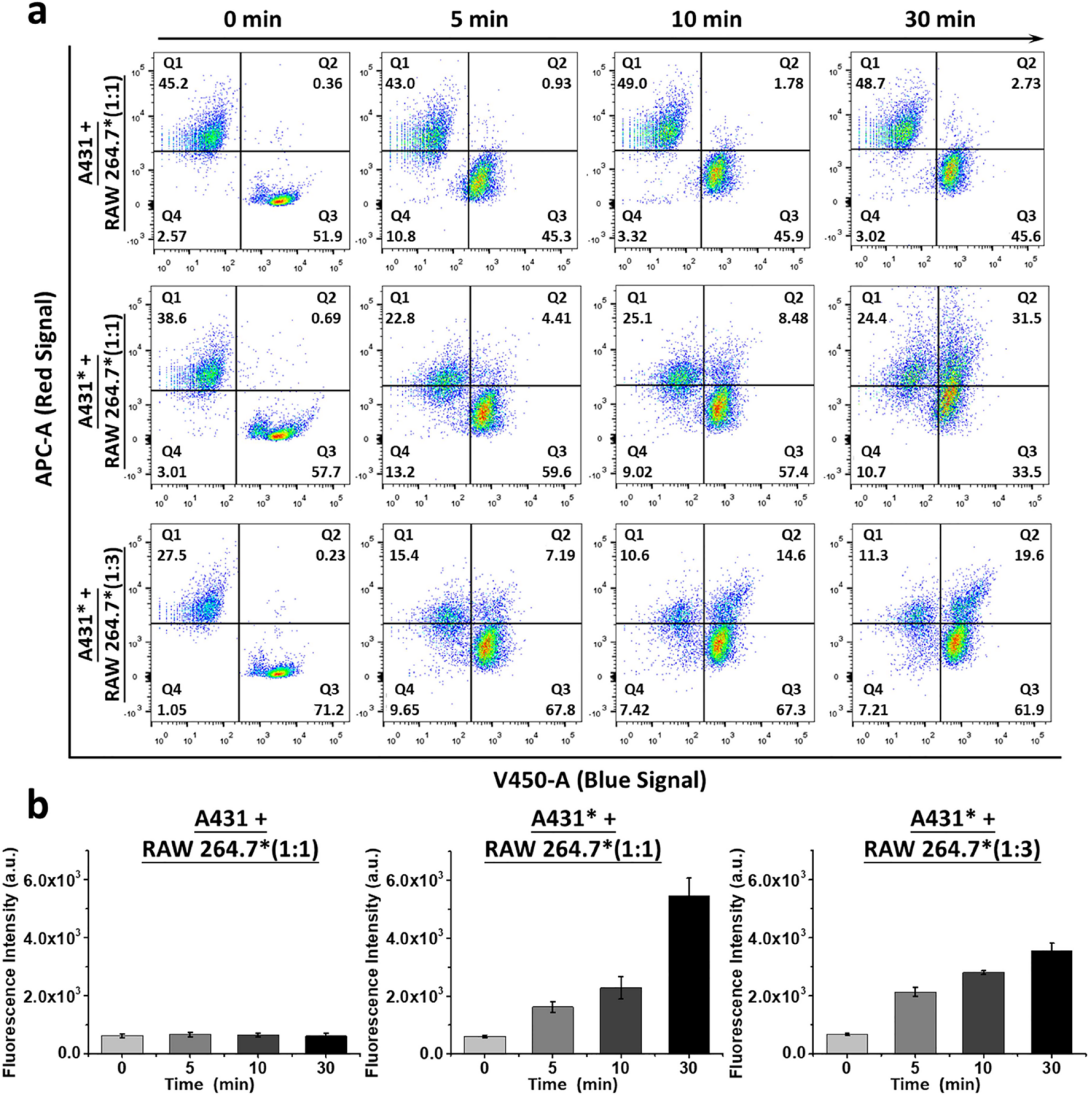
(a) Flow cytometric analysis of the cell assemblies of native A431 and RAW 264.7* cells (1:1) and A431* cells (treated with 40 μM of **Mal-BCN**) and RAW 264.7* cells (1:1 or 1:3). The cells were cocultured for different periods of time (from 0 to 30 min). (b) Change in fluorescence intensity of the activated **Mal-TzBDP** under these conditions. Data are reported as the mean ± SD of three independent experiments.

To provide further evidence of the bioorthogonal coupling of the two kinds of cells, the time-dependent connecting efficiency was also studied. Similarly, the native A431 or A431* (treated with 40 μM of **Mal-BCN**) cells were cocultured with RAW 264.7* cells in 1:1 or 1:3 ratio for different periods of time (from 0 to 30 min), and then they were subjected to flow cytometric analysis (Figure 5). As expected, both the cell population in Q2 and the fluorescence intensity of the activated **Mal-TzBDP** remained essentially unchanged for the 1:1 mixture of native A431 and RAW 264.7* cells over a period of 30 min. By replacing the native A431 cells with A431* cells, while the cell population in Q2 increased gradually to 32%, the fluorescence intensity of the activated **Mal-TzBDP** also increased gradually by ca. 10-fold after 30 min. The same trend was observed for the 1:3 mixture, though the extent of increase was smaller under this condition. These results further demonstrated that the cell–cell connecting efficiency and the fluorescence intensity of the activated **Mal-TzBDP** are directly proportional, making the latter a quantitative indicator for the iEDDA-promoted cell–cell interactions.

**Figure 6:**
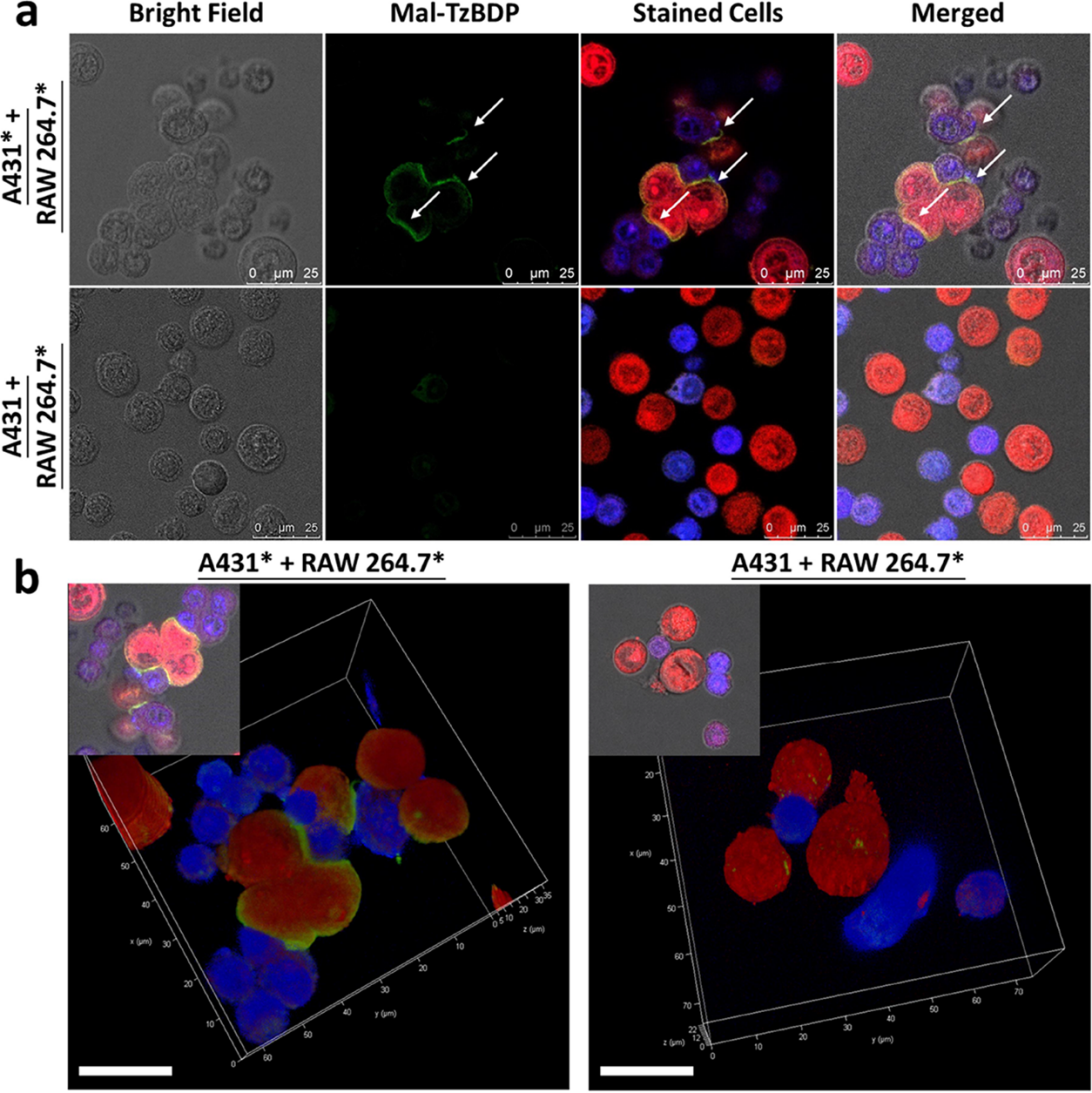
(a) Bright field, fluorescence (due to the activated **Mal-TzBDP** or the stained cells), and the merged confocal images of the cell assemblies of A431* cells (treated with 40 μM of **Mal-BCN**) or the unmodified A431 cells and RAW 264.7* cells cocultured in 1:1 ratio for 30 min. The A431 and RAW 264.7 cells were stained with CellTracker Red CMTPX Dye and CellTrace Violet to give red and blue fluorescence, respectively. The white arrows indicate the green fluorescence of the activated **Mal-TzBDP** appeared at the cell–cell contact sites. (b) Three-dimensional confocal images of these cell mixtures. The inset in each figure shows the corresponding two-dimensional confocal image. Scale bar: 20 μm.

Confocal microscopy was then used to further examine the binding interactions between the two kinds of cells. As shown in Figure 6a, after coculturing A431* and RAW 264.7* (1:1) cells for 30 min, cell assemblies were observed. Interestingly, a clear green fluorescence signal (indicated by a white arrow) due to the activated **Mal-TzBDP** appeared at the interface of two interacting cells. In contrast, when the unmodified A431 cells were used instead, all the cells remained discrete. The same results were observed in the low-magnification fluorescence images of these mixtures (Figure S9), which showed a larger population of cells. To take a closer look at the cell–cell contact, we also captured the three-dimensional images of the cells. As shown in Figure 6b and Videos S1 and S2 in the Supporting Information, for the mixture of A431* and RAW 264.7* cells, the green fluorescence due to the activated **Mal-TzBDP** appeared at almost all the contact sites. In contrast, for the mixture of unmodified A431 cells and RAW 264.7* cells, the absence of green fluorescence at the cell interfaces indicated that the immobilized **Mal-TzBDP** was not activated due to the absence of BCN-based activator. Even though the two kinds of cells might come into contact randomly during migration, they were not covalently linked. These results clearly showed that this bioorthogonal strategy can also be utilized to visualize the physical cell–cell interactions promoted by the iEDDA reaction by confocal fluorescence microscopy.

### Phagocytosis of Tumor Cells by Macrophages

**Figure 7:**
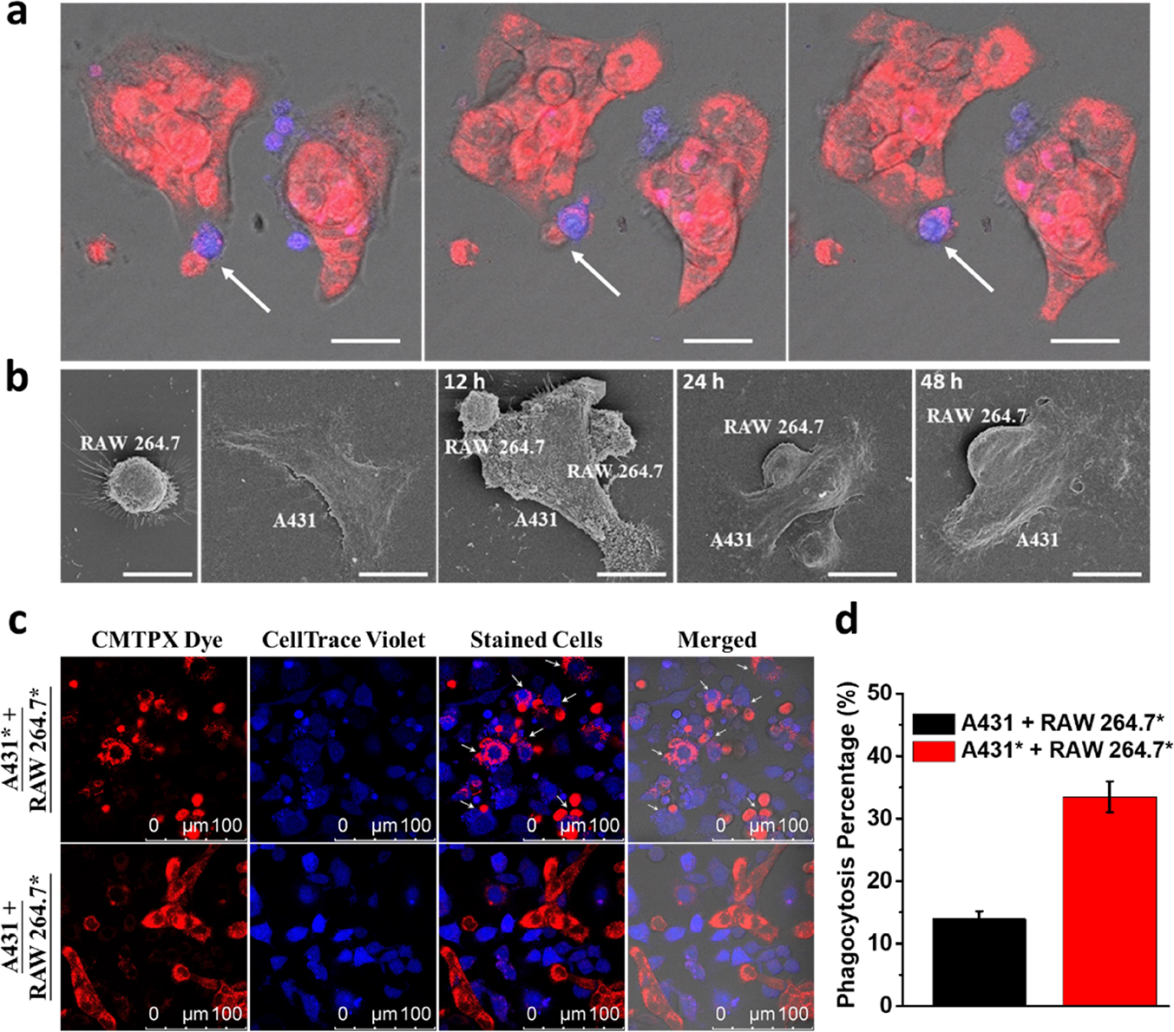
(a) Selected time-lapsed confocal images taken when the engulfment occurred. Scale bar: 20 μm. (b) Representative SEM images of A431* and RAW 264.7* cells, as well as their 1:1 mixture after being cocultured for 30 min, followed by incubation in the culture medium for a further 12, 24, and 48 h. Scale bar: 15 μm. (c) Representative confocal images of CMTPX-stained A431* or A431 cells phagocytized by CellTrace Violet-stained RAW264.7* cells after coculturing for 30 min, followed by incubation in the culture medium for 24 h. (d) Corresponding quantified phagocytosis events, calculated as the number of A431*/A431-phagocytized macrophages divided by the total number of macrophages ×100%. Data are reported as the mean ± SD of three independent experiments.

Finally, the phagocytosis of A431* cells by the physically connected RAW 264.7* cells was studied. After surface adhesion of the two kinds of cells via iEDDA reaction, the cells were further incubated in the culture medium for 12 h to enhance the phagocytic ability of RAW264.7* cells and to allow the attachment of all the cells on the confocal dish. The phagocytosis process was then monitored by confocal fluorescence microscopy over a period of 12 h. A video recording the process is given in Video S3, while Figure 7a shows selected time-lapsed confocal images taken when the engulfment occurred. As these RAW 264.7 macrophage cells were not activated,^59^ their phagocytic effect was relatively weak. Nevertheless, the images clearly show the partial engulfment of the red-fluorescent A431* cells by the blue-fluorescent RAW 264.7* cells during the course. The cell–cell interactions were further investigated by scanning electron microscopy (SEM). As shown in Figure 7b, the morphologies of A431* and RAW 264.7* cells were distinct, and direct contact of the two kinds of cells could be clearly observed after postincubation of their 1:1 mixture for 12 h. After coculturing for a further 12 h, the RAW 264.7* cells started to merge with the A431* cells, and this process was essentially completed after a further 24 h. These results suggested that the bioorthogonally triggered cell adhesion could promote phagocytosis of cancer cells by macrophages.

To promote the phagocytosis, RAW 264.7 cells were first activated upon incubation with lipopolysaccharide (LPS) (100 ng mL^–1^) for 24 h according to the established protocol.^60,61^ The cells were then treated with TCEP (1 mM) for 30 min and then with **Mal-TzBDP** (8 μM) for a further 30 min. Subsequently, these LPS-activated RAW 264.7* cells were cocultured with A431* cells or the unmodified counterpart in a 1:1 ratio. After coculturing for 30 min, the cells were incubated in the culture medium for a further 12 h. As shown in Figure S10, aggregates of RAW 264.7* and A431* cells were clearly observed, while the cells remained discrete when the unmodified A431 cells were used. This observation was similar to that for the non-LPS-treated RAW 264.7* (Figure S9), showing that the LPS treatment did not affect the interactions between RAW 264.7* and A431* cells.

To monitor the phagocytosis process, a confocal microscope was used to capture the fluorescence images after coculturing for 30 min, followed by incubation in the culture medium for 24 h. The results as shown in Figure 7c clearly demonstrated that the preactivated RAW 264.7* cells could efficiently phagocytize and digest A431* cells. The fluorescence of CMTPX dye dispersed in approximately 34% of the RAW 264.7* cells at the end of the experiment. In contrast, only about 15% of RAW 264.7* cells phagocytized the unmodified A431 cells (Figure 7d). These findings further indicated that the presence of the bioorthogonal components on the cell surfaces facilitated and prolonged the interactions between the two kinds of cells, and by preactivating the macrophage cells, the phagocytosis process could be greatly promoted.

## Conclusions

In summary, we have designed and synthesized a tetrazine-caged BODIPY-based fluorophore and a BCN both substituted with a terminal maleimide group via a hydrophilic peptide sequence. These two conjugates can be used to modify the surface of macrophage (RAW 264.7) and cancer (HT29, HeLa, and A431) cells efficiently via maleimide–thiol conjugation. Upon encountering BCN moieties in solution, inside cancer cells, and on the cell surface, the tetrazine-caged BODIPY can be activated through bioorthogonal coupling to restore its fluorescence emission. After modification of the cell surface with these complementary bioorthogonal components, the two kinds of cells can link up with each other readily via iEDDA reaction, and the intercellular interactions can be detected by the fluorescence of the activated BODIPY at the interface of the interacting cells, of which the intensity is proportional to the connecting efficiency of the cells. The promotion and detection of cell–cell interactions have been demonstrated using flow cytometry and confocal microscopy. The interactions between the RAW 264.7 murine macrophage cells and A431 cancer cells result in enhanced phagocytosis as revealed by confocal microscopy and SEM. This study shows that this bioorthogonal turn-on strategy can promote and facilitate the detection of cell–cell interactions.

While the current study primarily concentrates on monitoring iEDDA-promoted intercellular interactions rather than native cell−cell interactions, it is anticipated that this strategy could be adapted to the latter by suitably reducing the spacer between the maleimide unit and the BCN/tetrazine moiety. Similar to the methodology of GRASP mentioned above,^22−24^ in which the two nonfluorescent split GFP fragments genetically fused to the interacting cell partners respectively combine to emit the GFP fluorescence when the cells come into close contact, the fluorogenicity of **Mal-TzBDP** can be adopted as a reporter for native cell–cell interactions if the bioorthogonal handles are optimally positioned on the cell surface. The visualization of physical cell–cell interactions enabled by this approach suggests that it may also be used to detect the location of synaptic partners and facilitate the synaptic mapping through modification of the extracellular membrane of different neurons with bioorthogonal activatable components. Moreover, it is envisaged that this strategy can also be extended for modification of T cells to enhance their recognition and migration to tumor cells, thereby overcoming their drawback of low affinity toward the tumor-associated antigens, which could largely promote the cell-based cancer immunotherapy. We hope that this study can stimulate further investigation in these endeavors.

## Supporting Information Available

Experimental details; change in fluorescence spectrum of **Mal-TzBDP** in the absence and presence of **BCN–OH** in PBS at pH 7.4 with time; confocal images of HT29 and HeLa cells after incubation with TCEP and **Mal-TzBDP** with or without further incubation with **BCN–OH**; synthesis of **GE11-BCN**; confocal images and flow cytometric data for the activation of **Mal-TzBDP** by **GE11-BCN** in HeLa cells; confocal images of A431 cells after sequential incubation with TCEP, **Mal-BCN**, and **Mal-TzBDP**, followed by incubation in neat medium for a further 1, 4, and 8 h, respectively; confocal and flow cytometric studies of the role of TCEP; cytotoxicity of TCEP; cytotoxicity of **Mal-TzBDP** and **Mal-BCN** toward RAW 264.7, A431, and HT29 cells; flow cytometric analysis of the assemblies of A431/A431* or HT29/HT29* and RAW 264.7* cells; confocal images of the assemblies of A431/A431* and RAW 264.7* (with or without pretreatment with LPS) cells; HPLC chromatograms, NMR spectra, and/or ESI mass spectra of all the new compounds (PDF)
Activation of **Mal-TzBDP** at the interface of interacting A431* and RAW 264.7* cells (MP4)
Three-dimensional confocal image of the cell assembly of A431 and RAW 264.7* cells (MP4)
Phagocytosis process monitored by confocal fluorescence microscopy over a period of 12 h (MP4)
