# Supplementary material for: Promotion and Detection of Cell–Cell Interactions through a Bioorthogonal Approach
Source: J Am Chem Soc. 2024 May 20;146(25):17334–47. doi: 10.1021/jacs.4c04317 (PMC11212048; doi:10.1021/jacs.4c04317)
Supplement: Supplementary file 1 — ja4c04317_si_001.pdf [file ja4c04317_si_001.pdf]

Supporting Information

Promotion and Detection of Cell-Cell Interactions Through a Bioorthogonal Approach

Evelyn Y. Xue,[†] Alan Chun Kit Lee,[‡,§] Kwan T. Chow,[¶] and Dennis K. P. Ng*[†]

[†] *Department of Chemistry, The Chinese University of Hong Kong, Shatin, N.T., Hong Kong, China. E-mail: dkpn@cuhk.edu.hk*

[‡] *School of Life Sciences, The Chinese University of Hong Kong, Shatin, N.T., Hong Kong, China*

[§] *Department of Applied Biology and Chemical Technology, The Hong Kong Polytechnic University, Kowloon, Hong Kong, China*

[¶] *Department of Biomedical Sciences, City University of Hong Kong, Kowloon, Hong Kong, China*

Contents

Experimental Section

Figure S1 Change in fluorescence spectrum of **Mal-TzBDP** (2 μM) in the (a) absence and (b) presence of **BCN-OH** (10 μM) in PBS at pH 7.4 over a period of 60 min (λ_{ex} = 488 nm).

Figure S2 Bright field, fluorescence, and the merged confocal images of HT29 and HeLa cells after incubation with TCEP (1 mM) for 30 min and then with **Mal-TzBDP** (4 μM) for 30 min with or without further incubation with **BCN-OH** (10 μM) for 30 min.

Scheme S1 Synthesis of the conjugate **GE11-BCN**.

Figure S3 (a) Bright field, fluorescence, and the merged confocal images of HeLa cells after incubation with TCEP (1 mM) for 30 min and then with **Mal-TzBDP** (4 μM) for 30 min with or without further incubation with **GE11-BCN** (10 μM) for 15 or 30 min. (b) Corresponding histograms showing the fluorescence intensities determined by flow cytometry. (c) Corresponding quantified fluorescence intensities determined by flow cytometry.

Figure S4 Bright field, fluorescence, and the merged confocal images of A431 cells after sequential incubation with TCEP (1 mM) for 30 min, **Mal-BCN** (10 μM) for 30 min, and **Mal-TzBDP** (4 μM) for 30 min, followed by incubation in neat medium for a further 1, 4, and 8 h, respectively.

Figure S5 (a) Confocal images of HT29 and RAW 264.7 cells after sequential incubation with **Mal-TzBDP** (4 μM) for 30 min and **Mal-BCN** (10 μM) for 30 min, as well

as A431 cells in reverse order. Flow cytometric results for (b) HT29, (c) RAW 264.7, and (d) A431 cells after the above treatments with or without pre-incubation with TCEP (1 mM) for 30 min. The control group represents the cells being incubated only with **Mal-TzBDP**. (e) Corresponding quantified fluorescence intensities determined by flow cytometry.

Figure S6 Cell viability of HT29, A431, and RAW 264.7 cells after incubation with TCEP (0-2 mM) for 30 min.

Figure S7 (a) Cell viability of RAW 264.7 cells after sequential incubation with TCEP (1 mM) for 30 min and different concentrations of **Mal-TzBDP** (0-40 μM) for 30 min. (b) Cell viability of A431 cells after sequential incubation with TCEP (1 mM) for 30 min and different concentrations of **Mal-BCN** (0-40 μM) for 30 min. (c) Cell viability of HT29 cells after sequential incubation with TCEP (1 mM) for 30 min and different concentrations of **Mal-BCN** (0-40 μM) for 30 min.

Figure S8 Flow cytometric analysis of different cell assemblies: (a) For native A431 or A431* cells (treated with 10, 20, or 40 μM of **Mal-BCN**) and RAW 264.7* cells co-cultured in 1:1 ratio for 30 min. (b) For native HT29 cells and RAW 264.7* cells co-cultured in 1:1 ratio for 30 min. (c) For HT29* and RAW 264.7* cells co-cultured in 1:1 ratio for 30 min. (d) Quantified fluorescence intensities of the activated **Mal-TzBDP** in the cell assemblies of (b) and (c).

Figure S9 Confocal images of the cell assemblies of A431* (treated with 40 μM of **Mal-BCN**) or the unmodified A431 cells and RAW 264.7* cells co-cultured in 1:1 ratio for 30 min.

Figure 10 Confocal images of the cell assemblies of A431* (treated with 40 μM of **Mal-BCN**) or the unmodified A431 cells and LPS-treated RAW 264.7* cells co-cultured in 1:1 ratio for 30 min, followed by incubation in the culture medium for 12 h.

Figure S11 ^{1}H and $^{13}C\{^{1}H\}$ NMR spectra of **7** in $CDCl_3$.

Figure S12 ^{1}H and $^{13}C\{^{1}H\}$ NMR spectra of **9** in $CDCl_3$.

Figure S13 ^{1}H and $^{13}C\{^{1}H\}$ NMR spectra of **10** in $CDCl_3$.

Figure S14 ^{1}H and $^{13}C\{^{1}H\}$ NMR spectra of **12** in $CDCl_3$.

Figure S15 ^{1}H and $^{13}C\{^{1}H\}$ NMR spectra of **14** in $CDCl_3$.

Figure S16 MALDI-TOF mass spectrum of **3**.

Figure S17 MALDI-TOF mass spectrum of **Mal-BCN**.

Figure S18 ESI mass spectrum of **7**.

Figure S19 ESI mass spectrum of **9**.

Figure S20 ESI mass spectrum of **10**.

Figure S21 ESI mass spectrum of **12**.

Figure S22 ESI mass spectrum of **14**.

Figure S23 MALDI-TOF mass spectrum of **Mal-TzBDP**.

Figure S24 MALDI-TOF mass spectrum of **GE11-BCN**.

Figure S25 HPLC chromatograms of (a) **3**, (b) **Mal-BCN**, (c) **Mal-TzBDP**, and (d) **GE11-BCN.**

Experimental Section

General

All the reactions were performed under an atmosphere of nitrogen. DMF and CH_2Cl_2 were dried using an INERT solvent purification system. All other solvents and reagents were used as received. Chromatographic purification was performed on silica gel (Macherey-Nagel 230-400 mesh) with the indicated eluent. Compounds **4**[R1] and **8**[R2] and the GE11 peptide[R3] were prepared as described.

^{1}H and $^{13}C\{^{1}H\}$ NMR spectra were recorded on a Bruker AVANCE III 400 spectrometer (^{1}H, 400 MHz; ^{13}C, 100.6 MHz) in $CDCl_3$. Spectra were referenced internally using the residual solvent (^{1}H: δ 7.26) or solvent (^{13}C: δ 77.2) resonance relative to $SiMe_4$. Matrix-assisted laser desorption/ionization time-of-flight (MALDI-TOF) mass spectra were recorded on a Bruker Daltonics Autoflex III spectrometer with α-cyano-4-hydroxycinnamic acid as the matrix. Electrospray ionization (ESI) mass spectra were recorded on a Thermo Finnigan MAT 95 XL mass spectrometer. SEM images were obtained using a Hitachi SU8010 cold-field scanning electron microscope. Steady-state fluorescence spectra were taken on a Horiba FluoroMax spectrofluorometer.

Reverse-phase high-performance liquid chromatography (HPLC) separation was performed on an Apollo-C18 column (5 μm, 4.6 mm × 150 mm) at a flow rate of 1 mL min^{-1} for analytical purpose or on a XBridge BEH300 Prep C18 column (5 μm, 10 mm × 250 mm) at a flow rate of 3 mL min^{-1} for preparative purpose, using a Waters system equipped with a Waters 1525 binary pump and a Waters 2998 photodiode array detector. The solvents used for HPLC analysis were of HPLC grade. The HPLC conditions were set as follows: solvent A =

0.1% TFA in acetonitrile; solvent B = 0.1% TFA in distilled water. The gradient was 100% B in the first 5 min, and then changed to 100% A in 35 min, maintained under this condition for 10 min, changed back to 100% B in 5 min, and finally kept under this condition for 10 min.

Preparation of 3

Resin **1** with the peptide sequence EEHRPEGGGGSK was synthesized manually using a modified Fmoc SPPS protocol with the commercially available *N*-α-Fmoc-protected amino acids as starting materials. The rink amide resin was used as the solid support, and HATU was used as the carboxyl group activating agent. The swelled resin was treated with piperidine (20%) in DMF for 20 min twice before being coupled with the first amino acid. The Fmoc-protected amino acid (4 equiv.), HATU (4 equiv.), and DIPEA (8 equiv.) were used for each coupling. After the final coupling, the *N*-terminal of the peptide resin was coupled with 6-maleimidohexanoic acid (**2**) (4 equiv.) overnight in the presence of HATU (4 equiv.) and DIPEA (8 equiv.). After being washed with DMF and CH_2Cl_2 twice, the peptide resin was treated with a mixture of TFA (95%), TIPS (2.5%), and H_2O (2.5%) (4 mL) for 2 h to detach the peptide from the resin and remove the protecting groups. The resin was removed by filtration and the filtrate was precipitated by the addition of cold diethyl ether. After centrifugation, the supernatant was removed. The white solid obtained was re-dissolved in DMF (0.5 mL) and then precipitated again by the addition of diethyl ether. This purification procedure was repeated twice, and then the product was further purified using HPLC by monitoring the absorbance at 220 nm. The HPLC yield was determined to be 96%, and the

purity of **3** was found to be >95% by HPLC analysis. MS (MALDI-TOF): *m/z* calcd for $C_{59}H_{90}N_{19}O_{23}$ $[M+H]^+$ 1432.645, found 1432.639.

Preparation of Mal-BCN

A mixture of **3** (10 mg, 7.0 μmol), **4** (4 mg, 12.7 μmol), and DIPEA (20 μL) in DMF (1 mL) was stirred at room temperature for 6 h. After evaporation, the residue was purified using HPLC by monitoring the absorbance at 220 nm to give a white solid (6.4 mg, 56%). The purity of the product was found to be >95% by HPLC analysis. MS (MALDI-TOF): *m/z* calcd for $C_{70}H_{101}N_{19}NaO_{25}$ $[M+Na]^+$ 1629.711, found 1629.746.

Preparation of 7

A solution of NaOH (0.4 g, 10 mmol) in water (1 mL) was added to a mixture of tetraethylene glycol (**5**) (43.4 g, 0.22 mol) and acrylonitrile (**6**) (10.0 g, 0.19 mol). The mixture was heated with stirring to approximately 60 °C within 20 min and then cooled to ambient temperature over a period of about 30 min. It was then stirred under this condition overnight to give a pale-yellow mixture, to which 1 M HCl was added slowly to adjust the pH value to 7. The resulting mixture was extracted with CH_2Cl_2 (50 mL x 2). The combined organic portion was washed with water (50 mL) and then dried over anhydrous Na_2SO_4. After evaporation in vacuo, the residue was purified by column chromatography with $CHCl_3$/MeOH (50:1, v/v) as the eluent to give **7** as a colorless oil (24.3 g, 52%). ^1H NMR: δ 3.57-3.74 (m, 18 H, OCH_2), 2.87 (br s, 1 H, OH), 2.60-2.64 (m, 2 H, CH_2CN). $^{13}C\{^1H\}$ NMR: δ 118.0, 72.8, 70.8, 70.6 (two overlapping

signals), 70.5, 70.4, 70.2, 66.0, 61.7, 18.9. HRMS (ESI): *m/z* calcd for $C_{11}H_{21}NNaO_5$ $[M+Na]^+$ 270.1312, found 270.1310.

Preparation of 9

To a 100 mL Schlenk tube equipped with a stirrer bar, **7** (7.0 g, 28.3 mmol), **8** (1.0 g, 5.6 mmol), $Zn(OTf)_2$ (1.0 g, 2.8 mmol), and $NH_2NH_2{\cdot}H_2O$ (14.1 g, 0.28 mol) were added. The vessel was sealed, and the mixture was stirred in an oil bath at 60 °C for 24 h. The mixture was then cooled to room temperature, and the seal was removed. A solution of $NaNO_2$ (7.6 g, 0.11 mol) in water (20 mL) was slowly added to the mixture, followed by slow addition of 1 M HCl, during which the mixture turned to bright red in color with gas evolved. Addition of 1 M HCl continued until the gas evolution ceased and the pH value reached ca. 3. The mixture was extracted with ethyl acetate (50 mL x 2). The combined organic phase was dried over anhydrous Na_2SO_4 and then evaporated to dryness under reduced pressure. The residue was purified by chromatography on silica gel with $CHCl_3$/MeOH (50:1, v/v) as the eluent to afford **9** as a red solid (0.82 g, 32%). ^1H NMR: δ 8.61 (d, *J* = 8.4 Hz, 2 H, ArH), 7.68 (d, *J* = 8.4 Hz, 2 H, ArH), δ 5.50 (s, 1 H, CHO_2), 4.15 (t, *J* = 6.4 Hz, 2 H, Tz-CH_2), 3.57-3.71 (m, 18 H, OCH_2), 3.37 (s, 6 H, OCH_3). $^{13}C\{^1H\}$ NMR: δ 168.1, 164.3, 142.8, 132.0, 128.0, 127.8, 102.5, 72.7, 70.7, 70.6 (two overlapping signals), 70.5, 70.3, 68.6, 61.8, 52.9, 35.4. HRMS (ESI): *m/z* calcd for $C_{21}H_{32}N_4NaO_7$ $[M+Na]^+$ 475.2163, found: 475.2162.

Preparation of 10

To a 50 mL round-bottomed flask equipped with a stirrer bar, a solution of **9** (0.60 g, 1.33 mmol) in THF (1.5 mL), HCl (12 M, 0.6 mL), and H_2O (1 mL) were added. After vigorous stirring at room temperature for 30 min, the mixture was mixed with brine (10 mL) and ethyl acetate (10 mL). The resulting mixture was extracted with ethyl acetate (20 mL x 2). The combine organic layer was washed with aqueous $NaHCO_3$ and brine, dried over anhydrous Na_2SO_4, and then evaporated in vacuo. The residue was purified by chromatography on silica gel with $CHCl_3$/MeOH (50:1, v/v) as the eluent to afford **10** as a red solid (0.44 g, 82%). ^{1}H NMR: δ 10.16 (s, 1 H, CHO). 8.79 (d, *J* = 8.4 Hz, 2 H, ArH), 8.11 (d, *J* = 8.4 Hz, 2 H, ArH), 4.17 (t, *J* = 6.4 Hz, 2 H, Tz-CH_2), 3.58-3.71 (m, 18 H, OCH_2). $^{13}C\{^1H\}$ NMR: δ 191.8, 168.6, 163.8, 139.0, 137.2, 130.5, 128.7, 72.7, 70.7 (two overlapping signals), 70.6, 70.5, 70.4, 68.5, 61.9, 35.5. HRMS (ESI): *m/z* calcd for $C_{19}H_{26}N_4NaO_6$ $[M+Na]^+$ 429.1745, found 429.1746.

Preparation of 12

To a 250 mL round-bottomed flask equipped with a stirrer bar, **10** (0.44 g, 1.1 mmol) and 2,4-dimethylpyrrole (**11**) (0.24 g, 2.5 mmol) were dissolved in CH_2Cl_2 (50 mL). Two drops of TFA were then added, and the mixture was stirred at room temperature for 6 h when TLC indicated that **10** was completely consumed. A solution DDQ (0.27 g, 1.2 mmol) in CH_2Cl_2 (20 mL) was then added, and the mixture was stirred for a further 2 h. The mixture was then treated with triethylamine (1 mL) for 5 min. After that, $BF_3{\cdot}Et_2O$ (1 mL) was added and the mixture was stirred for a further 2 h. The dark brown mixture was then washed with water (50 mL) and brine (50 mL), dried over anhydrous $MgSO_4$, and evaporated under reduced pressure. The crude product was purified by chromatography on silica gel with $CHCl_3$/MeOH (50:1, v/v) as

the eluent to afford **12** as a red solid (0.20 g, 30%). ^{1}H NMR: δ 8.75 (d, *J* = 8.0 Hz, 2 H, ArH), δ 7.55 (d, *J* = 8.0 Hz, 2 H, ArH), 6.00 (s, 2 H, pyrrole-H), 4.16 (t, *J* = 6.4 Hz, 2 H, Tz-CH_2), 3.58-3.72 (m, 18 H, CH_2O), 2.56 (s, 6 H, CH_3), 1.44 (s, 6 H, CH_3). $^{13}C\{^1H\}$ NMR: δ 168.4, 163.9, 156.1, 143.0, 140.3, 139.6, 132.7, 131.1, 129.3, 128.7, 121.6, 72.7, 70.6, 70.5, 70.4, 70.3, 68.6, 68.1, 61.8, 53.6, 35.4, 25.7, 14.7. HRMS (ESI): *m/z* calcd for $C_{31}H_{39}BF_2N_6NaO_5$ $[M+Na]^+$ 647.2941, found 647.2935.

Preparation of 14

A mixture of **12** (20 mg, 32 μmol), **13** (8 mg, 38 μmol), and pyridine (20 μL) in CH_2Cl_2 (10 mL) was stirred at room temperature for 4 h. The solvent was then evaporated under reduced pressure. The residue was purified by column chromatography on silica gel with $CHCl_3/CH_3OH$ (30:1, v/v) as the eluent to afford **14** as a red solid (17 mg, 67%). ^{1}H NMR: δ 8.75 (d, *J* = 8.4 Hz, 2 H, ArH), 8.27 (d, *J* = 9.2 Hz, 2 H, ArH), 7.56 (d, *J* = 8.4 Hz, 2 H, ArH), δ 7.38 (d, *J* = 9.2 Hz, 2 H, ArH), 6.00 (s, 2 H, pyrrole-H), 4.43-4.45 (m, 2 H, OCH_2), 4.16 (t, *J* = 6.4 Hz, 2 H, Tz-CH_2), 3.80-3.82 (m, 2 H, OCH_2), 3.61-3.70 (m, 14 H, OCH_2), 2.57 (s, 6 H, CH_3), 1.45 (s, 6 H, CH_3). $^{13}C\{^1H\}$ NMR: δ 168.4, 164.0, 156.2, 155.6, 152.6, 145.5, 143.0, 140.3, 139.7, 132.7, 131.1, 129.4, 128.8, 125.4, 121.9, 121.7, 70.8, 70.7, 70.6, 70.5, 68.7, 68.6, 68.4, 35.5, 14.8. HRMS (ESI): *m/z* calcd for $C_{38}H_{42}BF_2N_7NaO_9$ $[M+Na]^+$ 812.3004, found 812.2997.

Preparation of Mal-TzBDP

A mixture of **14** (5 mg, 6.3 µmol), **3** (9 mg, 6.3 µmol), and DIPEA (20 µL) in DMF (1 mL) was stirred at room temperature for 4 h. After evaporation, the residue was purified by HPLC to give a red solid (4.8 mg, 36%). The purity of the product was found to be >95% by HPLC analysis. MS (MALDI-TOF): *m/z* calcd for $C_{91}H_{126}BF_2N_{25}NaO_{29}$ $[M+Na]^+$ 2104.912, found 2104.900.

Preparation of GE11-BCN

A mixture of GE11 peptide (10 mg, 6.3 µmol), **4** (5 mg, 6.3 µmol), and DIPEA (20 µL) in DMF (1 mL) was stirred at room temperature for 6 h. After evaporation, the residue was purified using HPLC by monitoring the absorbance at 488 nm to give a white solid (5.9 mg, 55%). The purity of the product was found to be >95% by HPLC analysis. MS (MALDI-TOF): *m/z* calcd for $C_{86}H_{109}N_{17}NaO_{21}$ $[M+Na]^+$ 1738.788, found 1738.899.

Fluorescence Analysis of the Activation of Mal-TzBDP by BCN-OH

The bioorthogonal reaction was performed in a 1 cm × 1 cm quartz cuvette. A stock solution of **Mal-TzBDP** in DMF (2 mM) was first prepared, which was diluted with PBS at pH 7.4 to give a 2 µM solution. Another stock solution of **BCN-OH** in DMF (20 mM) was also prepared. An aliquot of this solution was then added to the PBS solution of **Mal-TzBDP** prepared above to make the final concentration of **BCN-OH** at 10 µM. The fluorescence spectrum of the mixture was recorded from 500 to 650 nm over a period of 60 min upon excitation at 488 nm.

LC-MS Analysis of the Activation of Mal-TzBDP by BCN-OH

Mal-TzBDP (2 μM) was dissolved in PBS at pH 7.4. The solution was treated with **BCN-OH** (10 μM) at 37 ºC. An aliquot of the mixture was collected at 10 and 60 min for LC-MS analysis. It was performed on a XSelect CSH C18 column (5 μm, 4.6 mm × 250 mm) at a flow rate of 0.8 mL min^{-1} using a Waters system equipped with a Waters Quaternary Solvent Manager-R, a Waters 2998 photodiode array detector, a Waters 2475 fluorescence detector, and a Waters single quadrupole detector 2. The solvents used were of LC-MS grade. The conditions used for the analysis were set as follows: solvent A = 0.01% formic acid in acetonitrile and solvent B = 0.01% formic acid in deionized water; gradient: 20% A + 80% B in the first 5 min, changed to 0% A + 100% B in 30 min, maintained under this condition for 10 min, and then changed to 100% A + 0% B in 15 min.

Cell Lines and Culture Conditions

HeLa human cervical carcinoma cells (ATCC® CCL-2™), A431 human squamous carcinoma cells (ATCC® CRL-1555™), and RAW 264.7 murine macrophage cells (ATCC® TIB-71™) were maintained in Dulbecco's modified Eagle medium (DMEM) (ThermoFisher Scientific, cat. no. 12100-046) supplemented with fetal bovine serum (FBS, 10%) (ThermoFisher Scientific, cat. no. 10270-106) and a penicillin-streptomycin solution (100 units mL^{-1} and 100 μg mL^{-1}, respectively). HT29 human colorectal adenocarcinoma cells (ATCC® HTB-38™) were maintained in Roswell Park Memorial Institute (RPMI) 1640 medium (Invitrogen, cat. no. 23400-021) supplemented with FBS (10%) and a penicillin-streptomycin solution (100 units mL^{-1} and 100 μg mL^{-1}, respectively). All cells were grown at 37 °C in a humidified 5% CO_2 atmosphere.

Activation of Mal-TzBDP by BCN-OH in Cells

Approximately 2 × 10^5 HT29 cells in 2 mL of the culture medium were incubated on confocal dishes of 35 mm diameter at 37 °C with 5% CO_2 overnight. After removing the medium, the cells were rinsed with PBS and then incubated with **Mal-TzBDP** (4 μM) for 30 min. The cells were then rinsed with PBS with or without further incubation with **BCN-OH** (10 μM) in a serum-free medium for 5 and 10 min, respectively. After being rinsed with PBS twice, the cells were examined and imaged with a Leica TCS SP8 high-speed confocal microscope equipped with a 488 nm argon laser. The BODIPY was excited at 488 nm and its fluorescence was monitored at 500-610 nm. The images were digitized and analyzed using a Leica Application Suite X software.

Cell Surface Modification with Mal-TzBDP

Approximately 2 × 10^5 HT29, HeLa, or RAW 264.7 cells in 2 mL of the culture medium were incubated on confocal dishes of 35 mm diameter at 37 °C with 5% CO_2 overnight. After removing the medium, the cells were rinsed with PBS and then incubated with 1 mM of TCEP for 30 min. After that, the cells were rinsed with PBS twice followed by incubation with **Mal-TzBDP** (4 μM) for 30 min. To verify whether **Mal-TzBDP** had been immobilized on the cell surface, the cells, after being rinsed with PBS twice, were further incubated with **GE11-BCN** (10 μM) for 15 or 30 min. The cells were rinsed with PBS again and then replenished with Hank's Balanced Salt Solution (HBSS) (1 mL) for confocal microscopic study as described above. For flow cytometric study, the cells were harvested by 0.25% trypsin-ethylenediaminetetraacetic

acid (0.4 mL). The activity of trypsin was quenched with the culture medium (0.5 mL), and the mixture was centrifuged at 1500 rpm for 3 min. The pellet was washed with PBS (1 mL) three times and then centrifuged. The cells were then suspended in HBSS (1 mL) and subjected to flow cytometric analysis using a BD FACSVerse flow cytometer (Becton Dickinson) with 10^4 cells counted in each sample. Cell fragments were excluded with forward and side-scatter gating to ensure that all the detected signals were originated from relatively intact cells. Signals from the activated **Mal-TzBDP** were recorded in Chanel-FITC. All experiments were performed in triplicate. The results were compared with those without the post-incubation with **GE11-BCN**.

Cell Surface Modification with Mal-BCN

Approximately 2×10^5 A431 cells in 2 mL of the culture medium were incubated on confocal dishes of 35 mm diameter at 37 °C with 5% CO_2 overnight. After removing the medium, the cells were rinsed with PBS and then incubated with 1 mM of TCEP for 30 min. After that, the cells were rinsed with PBS twice with or without further incubation with **Mal-BCN** (10 µM) for 30 min. To verify whether **Mal-BCN** had been immobilized on the cell surface, the cells, after being rinsed with PBS twice, were further incubated with **Mal-TzBDP** (4 µM) for 30 min. The cells were rinsed with PBS again and then immersed in HBSS (1 mL) for confocal microscopic and flow cytometric studies as described above.

Study of Photocytotoxicity

Approximately 1×10^4 HT29, A431, and RAW 264.7 cells per well in the culture medium were

inoculated in 96-well plates and incubated at 37 °C with 5% CO_2 overnight. The cells were first treated with TCEP (1 mM) for 30 min. After being rinsed with PBS for three times, the cells were further incubated with **Mal-TzBDP** or **Mal-BCN** at different concentrations for 30 min. The cells were then rinsed with PBS for three times and refed with 100 µL of the culture medium. Cell viability was determined by means of a colorimetric MTT assay as described previously.[R4]

Click-Induced Cell-Cell Interactions

A431 and HT29 cells at a density of 2×10^6 cells mL^{-1} were suspended in a CellTracker Red CMTPX Dye (2 µM) solution for 30 min. After being rinsed with PBS twice, the cells were further modified with **Mal-BCN** (10, 20, or 40 µM for A431 cells; 40 µM for HT29 cells) as described above. Similarly, RAW 264.7 cells at a density of 2×10^6 cells mL^{-1} were suspended in a CellTrace Violet (4 µM) solution for 30 min and then modified with **Mal-TzBDP** (8 µM) as described above. The native and **Mal-BCN**-modified A431 or HT29 cells were then co-cultured with the **Mal-TzBDP**-modified RAW 264.7 cells in a 1:1 or 1:3 ratio. The cell mixtures were shaken for 30 min at 37 °C in a thermostatic oscillator. The cells were then suspended in the culture medium and examined with a Leica TCS SP8 high-speed confocal microscope. Lasers at 405, 488, and 532 nm were used to excited CellTrace Violet, the activated **Mal-TzBDP**, and CellTracker Red CMTPX Dye, respectively. The images were digitized and analyzed using a Leica Application Suite X software. For quantification of the cell-cell connecting efficiency, flow cytometry was used to determine the percentages of discrete and assembled cells. All the assembled systems were diluted to fourfold series with

PBS. Cell fragments were excluded with forward and side-scatter gating to ensure that all the detected signals were originated from relatively intact cells. Signals from CellTrace Violet were recorded in Chanel V-450A, while signals from CellTracke Red CMTPX Dye were recorded in Chanel APC-A. The flow cytometry diagrams presented were obtained from a population of 1×10^4 cells.

Time-Lapsed Confocal Imaging of Phagocytosis

The A431 cells, which had been treated with 40 µM of **Mal-BCN**, and the **Mal-TzBDP**-modified RAW 264.7 cells were co-cultured in 1:1 ratio for 30 min as described above. The cell mixture was then incubated in the culture medium for a further 12 h, and then the cell-cell interactions were monitored with a Leica TCS SP8 high-speed confocal microscope over a period of 12 h. Time-lapsed images were captured at every 15 min interval.

SEM Measurements

After the co-culturing of A431* and RAW 264.7* cells (1:1) for 30 min as described above, the cells were incubated on clean glass slices at 37 °C with 5% CO_2 for 12, 24, and 48 h, respectively. The medium was then removed. The cells, after being rinsed with Sorensen's phosphate buffer (SPB) (0.1 M at pH 7.2), were immediately fixed with a 2.5% glutaraldehyde solution in SPB at room temperature for 30 min. The cells were then rinsed with SPB for 3 times (10 min each) and fixed in 1% osmium tetroxide for 30 min. After that, the cells were washed with distilled water for 3 times (10 min each) and then dehydrated with a graded ethanol series: 80% (10 min), 90% (10 min), 95% (15 min) for two times, and finally 100% (15 min)

for two times, before being dried in a critical point dryer. The specimens were mounted on specimen stubs and then coated with gold-palladium on a sputter coater before being examined under a scanning electron microscope.

Study of Phagocytosis Efficiency of Activated RAW 264.7*

RAW 264.7 cells were first treated with lipopolysaccharide (LPS) at a concentration of 100 ng mL^{-1} for 24 h to activate their phagocytic activity. After being rinsed with PBS twice, the cells at a density of 2×10^6 cells mL^{-1} were suspended in a CellTrace Violet (4 μM) solution for 30 min and then modified with **Mal-TzBDP** (8 μM) as described above. Similarly, A431 cells at a density of 2×10^6 cells mL^{-1} were suspended in a CellTracker Red CMTPX Dye (2 μM) solution for 30 min and then modified with **Mal-BCN** (40 μM) as described above. Subsequently, the modified RAW 264.7* cells were co-cultured with A431* cells or the unmodified A431 cells in a 1:1 ratio for 30 min, followed by further incubation in the culture medium for a further 24 h. The phagocytosis of A431*/A431 cells by RAW 264.7* was monitored with a Leica TCS SP8 high-speed confocal microscope.

References

(R1) Dommerholt, J.; Schmidt, S.; Temming, R.; Hendriks, L. J. A.; Rutjes, F. P. J. T.; van Hest, J. C. M.; Lefeber, D. J.; Friedl, P.; van Delft, F. L. Readily accessible bicyclononynes for bioorthogonal labeling and three-dimensional imaging of living cells. *Angew. Chem. Int. Ed.* **2010**, *49*, 9422–9425.

(R2) Flamigni, L.; Ventura, B.; Rasior, M.; Becherer, T.; Langhals, H.; Gryko, D. T. New

and efficient arrays for photoinduced charge separation based on perylene bisimide and corroles. *Chem. Eur. J.* **2008**, *14*, 169–183.

(R3) Guo, X.; Wong, R. C. H.; Zhou, Y.; Ng, D. K. P.; Lo, P.-C. A novel distyryl boron dipyrromethene with two functional tags for site-specific bioorthogonal photosensitisation towards targeted photodynamic therapy. *Chem. Commun.* **2019**, *55*, 13518–13521.

(R4) Xue, E. Y.; Shi, W.-J.; Fong, W.-P.; Ng, D. K. P. Targeted delivery and site-specific activation of β-cyclodextrin-conjugated photosensitizers for photodynamic therapy through a supramolecular bio-orthogonal approach. *J. Med. Chem.* **2021**, *64*, 15461–15476.

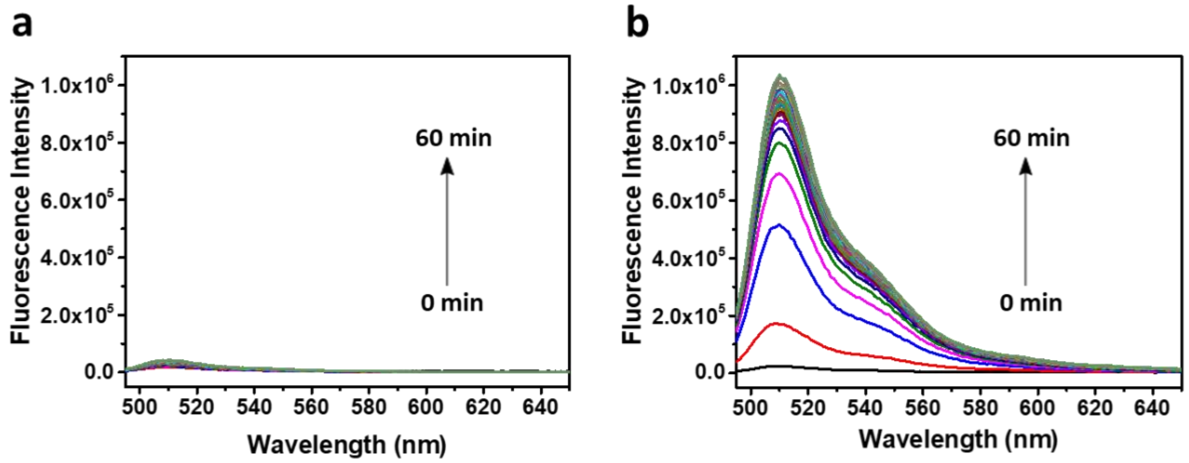

Figure S1. Change in fluorescence spectrum of **Mal-TzBDP** (2 μM) in the (a) absence and (b) presence of **BCN-OH** (10 μM) in PBS at pH 7.4 over a period of 60 min (λ_{ex} = 488 nm).

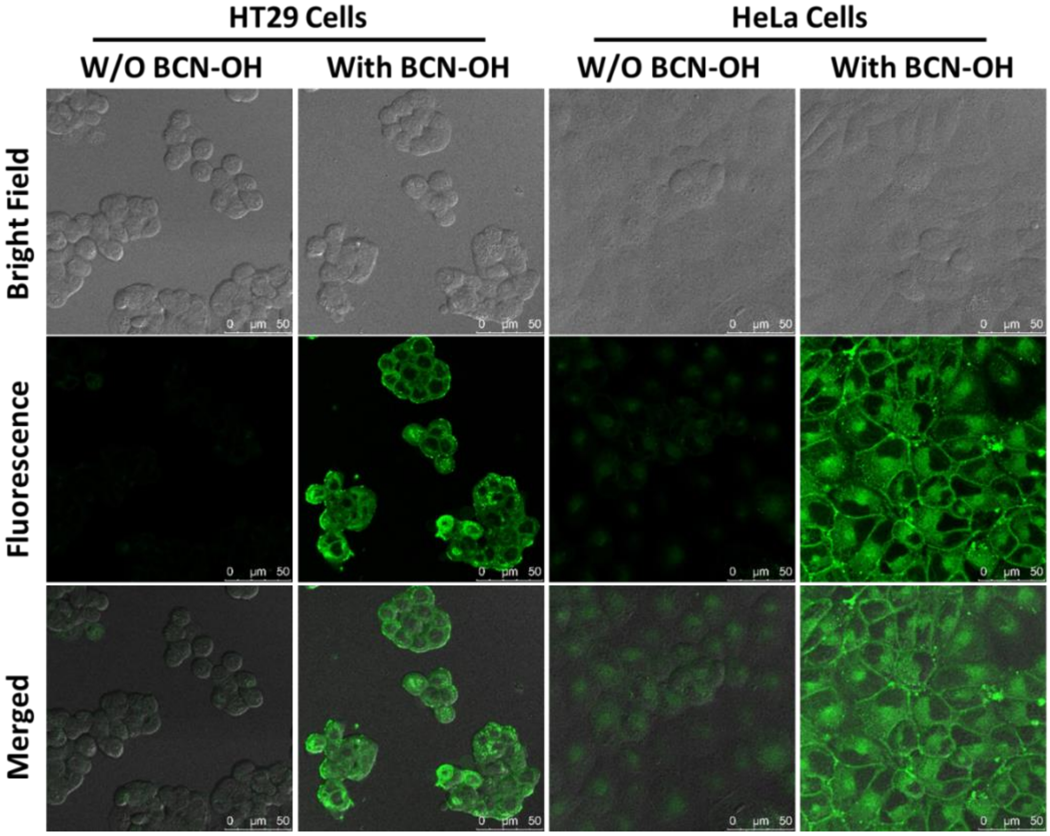

Figure S2. Bright field, fluorescence, and the merged confocal images of HT29 and HeLa cells after incubation with TCEP (1 mM) for 30 min and then with **Mal-TzBDP** (4 μM) for 30 min with or without further incubation with **BCN-OH** (10 μM) for 30 min.

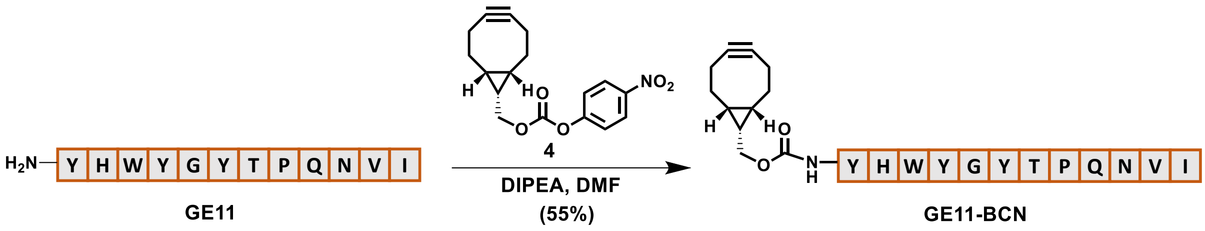

Scheme S1. Synthesis of the conjugate **GE11-BCN**.

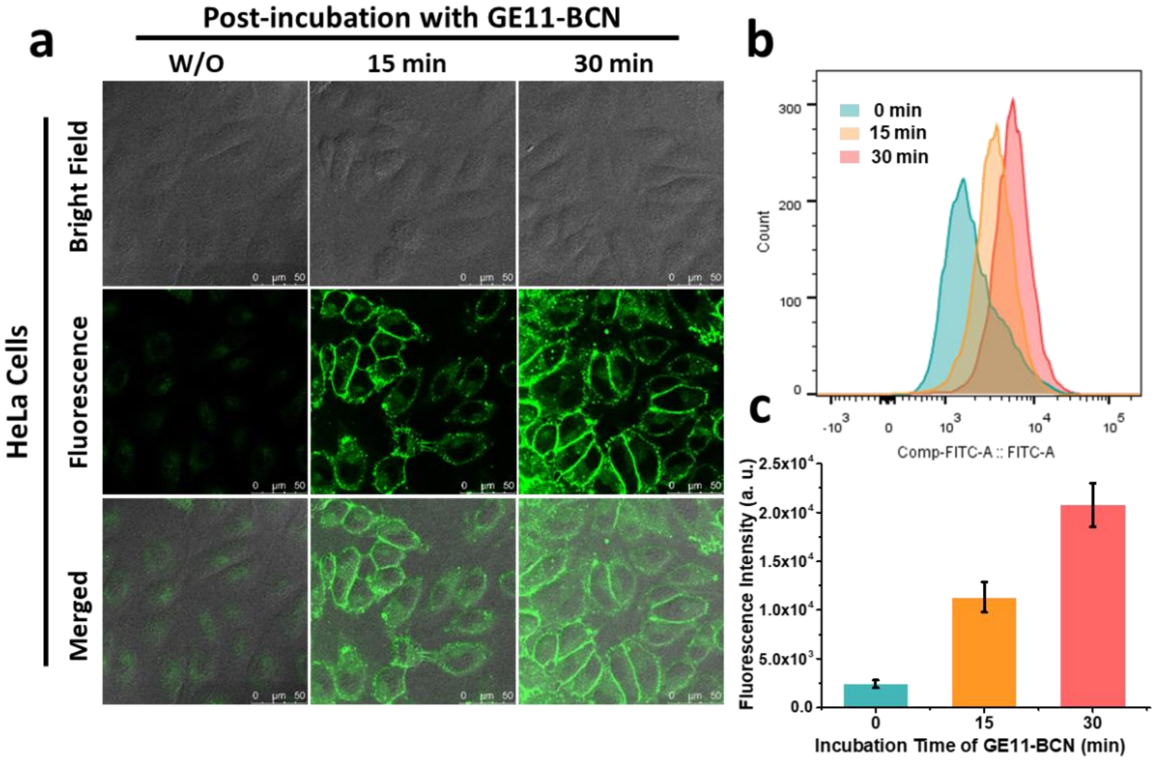

Figure S3. (a) Bright field, fluorescence, and the merged confocal images of HeLa cells after incubation with TCEP (1 mM) for 30 min and then with **Mal-TzBDP** (4 μM) for 30 min with or without further incubation with **GE11-BCN** (10 μM) for 15 or 30 min. (b) Corresponding histograms showing the fluorescence intensities determined by flow cytometry. (c) Corresponding quantified fluorescence intensities determined by flow cytometry. Data are expressed as the mean ± SD of three independent experiments.

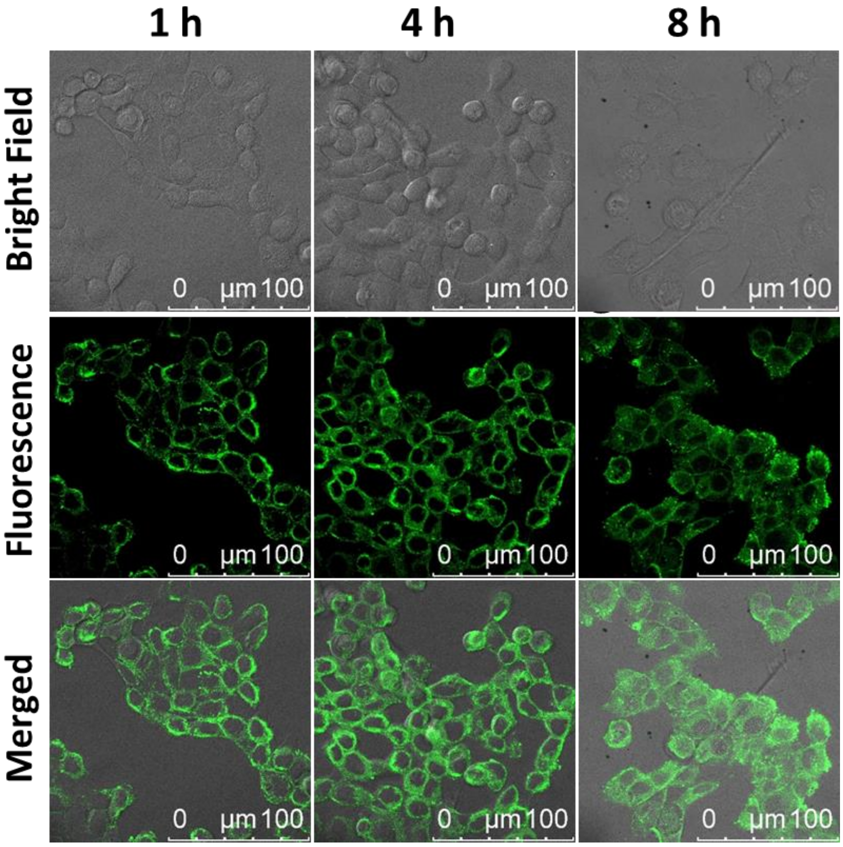

Figure S4. Bright field, fluorescence, and the merged confocal images of A431 cells after sequential incubation with TCEP (1 mM) for 30 min, **Mal-BCN** (10 μM) for 30 min, and **Mal-TzBDP** (4 μM) for 30 min, followed by incubation in neat medium for a further 1, 4, and 8 h, respectively.

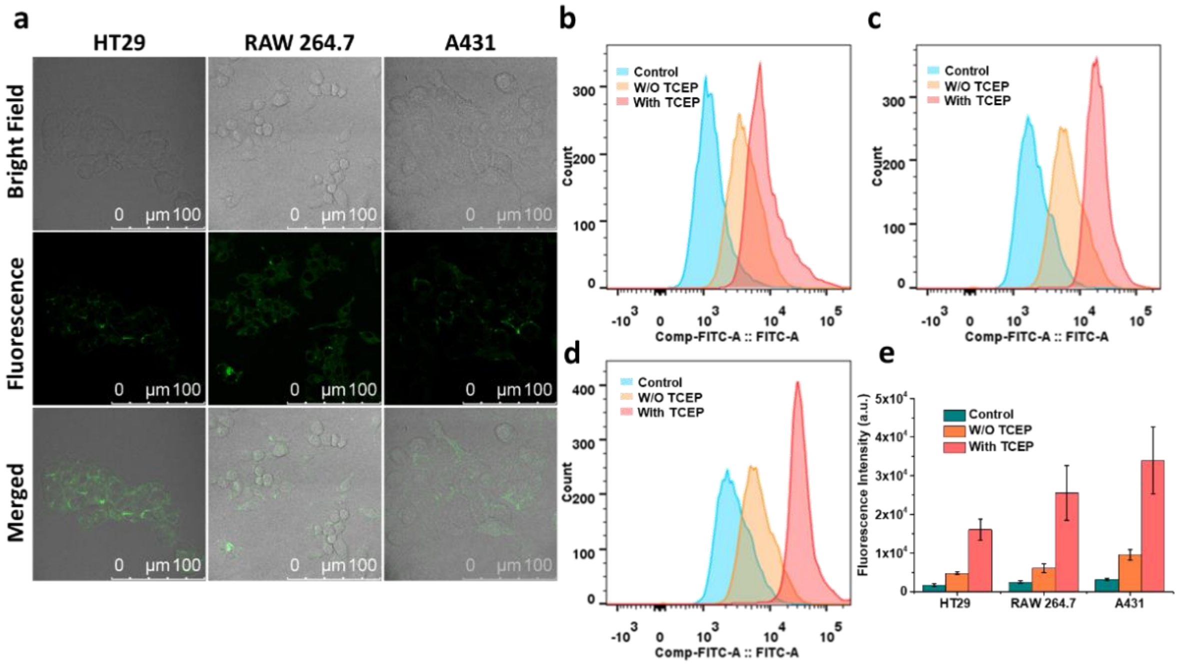

Figure S5. (a) Confocal images of HT29 and RAW 264.7 cells after sequential incubation with **Mal-TzBDP** (4 μM) for 30 min and **Mal-BCN** (10 μM) for 30 min, as well as A431 cells in reverse order. Flow cytometric results for (b) HT29, (c) RAW 264.7, and (d) A431 cells after the above treatments with or without pre-incubation with TCEP (1 mM) for 30 min. The control group represents the cells being incubated only with **Mal-TzBDP**. (e) Corresponding quantified fluorescence intensities determined by flow cytometry. Data are reported as the mean ± SD of three independent experiments.

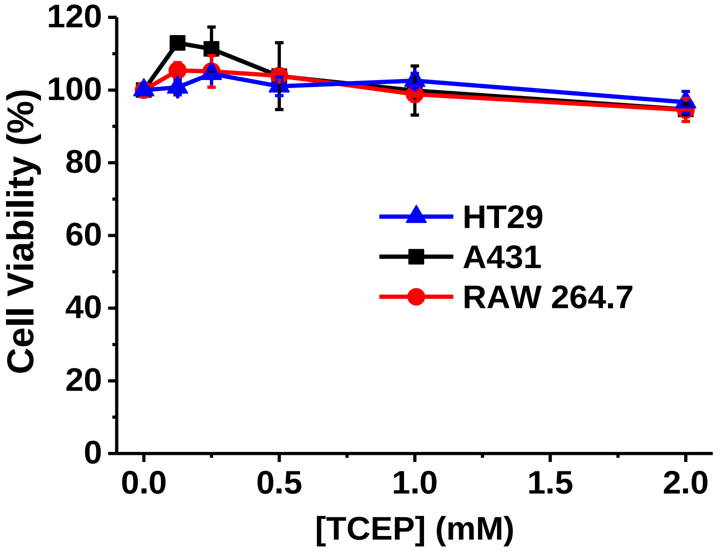

Figure S6. Cell viability of HT29, A431, and RAW 264.7 cells after incubation with TCEP (0-2 mM) for 30 min. Data are expressed as the mean ± standard error of the mean of three independent experiments, each performed in quadruplicate.

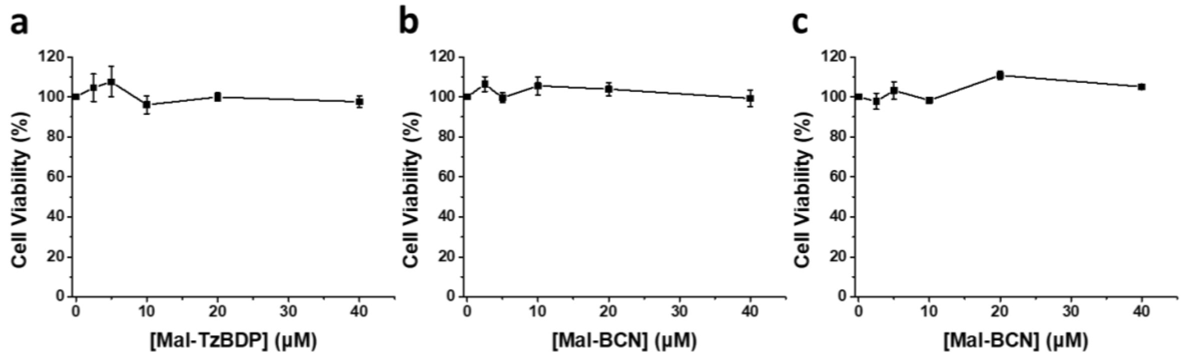

Figure S7. (a) Cell viability of RAW 264.7 cells after sequential incubation with TCEP (1 mM) for 30 min and different concentrations of **Mal-TzBDP** (0-40 μM) for 30 min. (b) Cell viability of A431 cells after sequential incubation with TCEP (1 mM) for 30 min and different concentrations of **Mal-BCN** (0-40 μM) for 30 min. (c) Cell viability of HT29 cells after sequential incubation with TCEP (1 mM) for 30 min and different concentrations of **Mal-BCN** (0-40 μM) for 30 min. Data are expressed as the mean ± standard error of the mean of three independent experiments, each performed in quadruplicate.

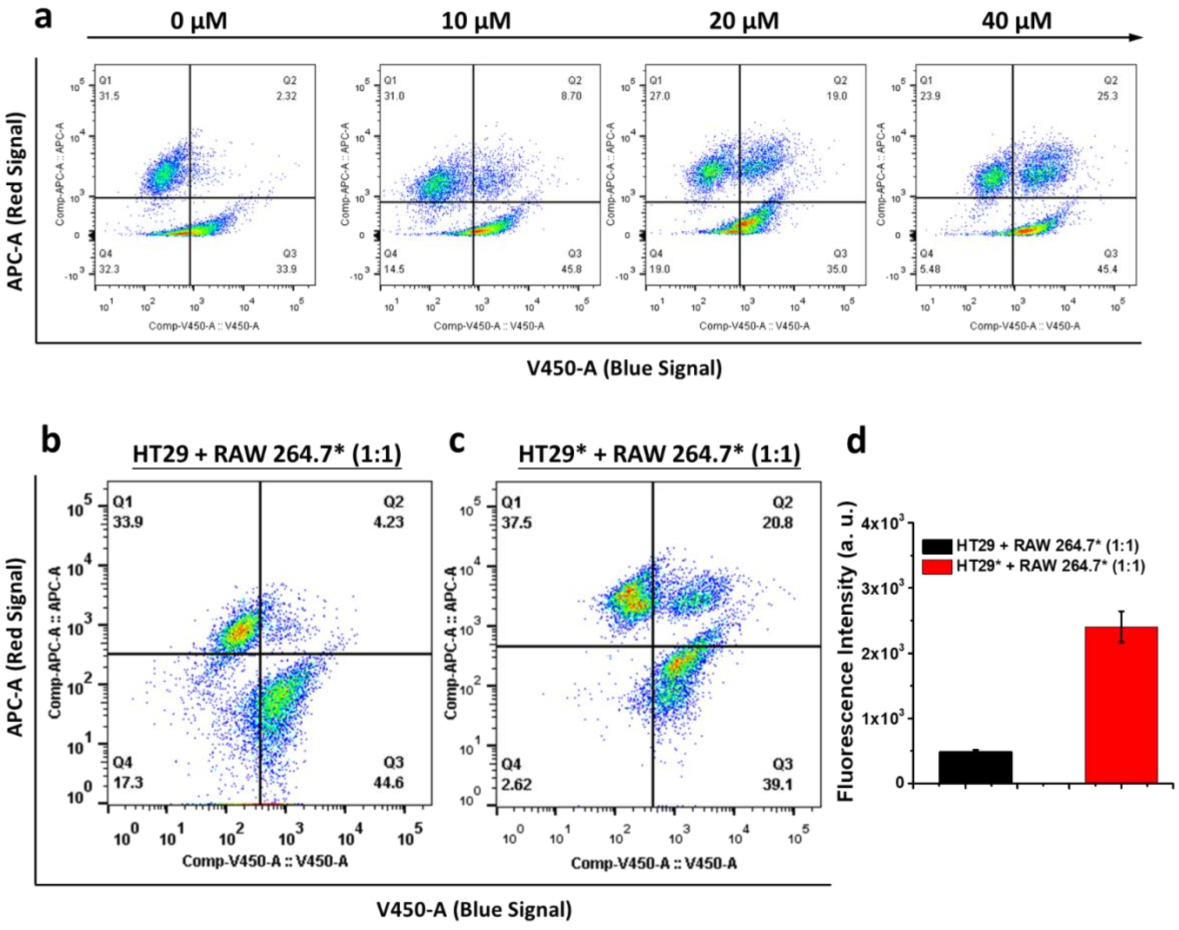

Figure S8. Flow cytometric analysis of different cell assemblies: (a) For native A431 or A431* cells (treated with 10, 20, or 40 μM of **Mal-BCN**) and RAW 264.7* cells co-cultured in 1:1 ratio for 30 min. (b) For native HT29 cells and RAW 264.7* cells co-cultured in 1:1 ratio for 30 min. (c) For HT29* and RAW 264.7* cells co-cultured in 1:1 ratio for 30 min. (d) Quantified fluorescence intensities of the activated **Mal-TzBDP** in the cell assemblies of (b) and (c). Data are reported as the mean ± SD of three independent experiments.

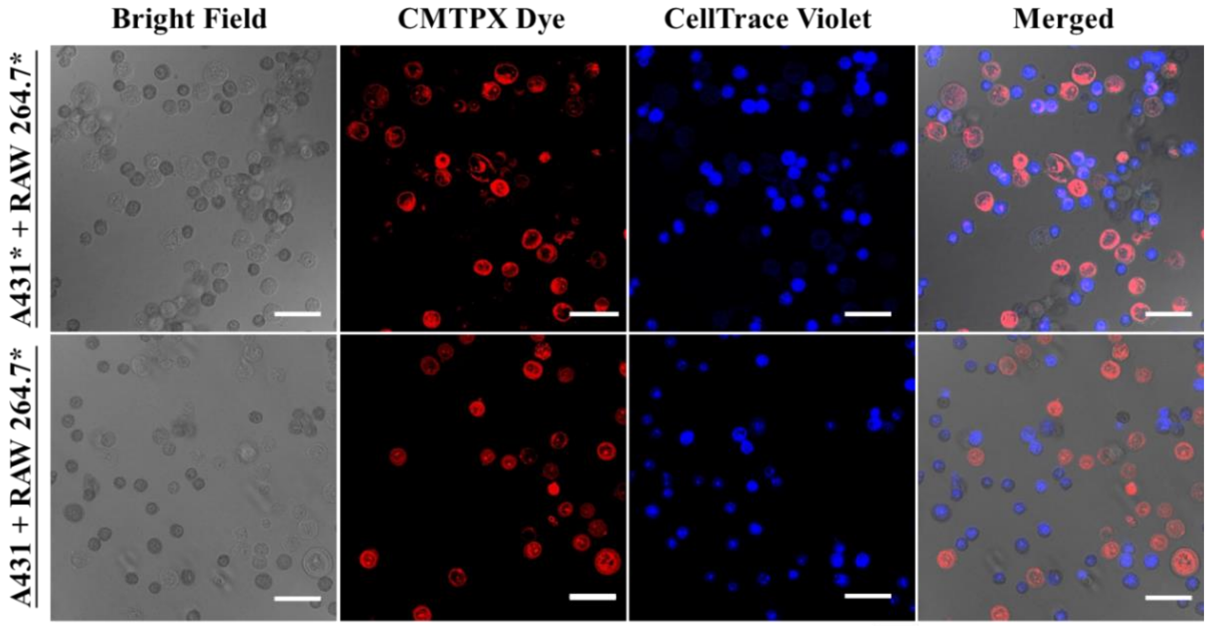

Figure S9. Confocal images of the cell assemblies of A431* (treated with 40 μM of **Mal-BCN**) or the unmodified A431 cells and RAW 264.7* cells co-cultured in 1:1 ratio for 30 min. The A431 and RAW 264.7 cells were stained with CellTracker Red CMTPX Dye and CellTrace Violet to give red and blue fluorescence, respectively. Scale bar: 40 μm.

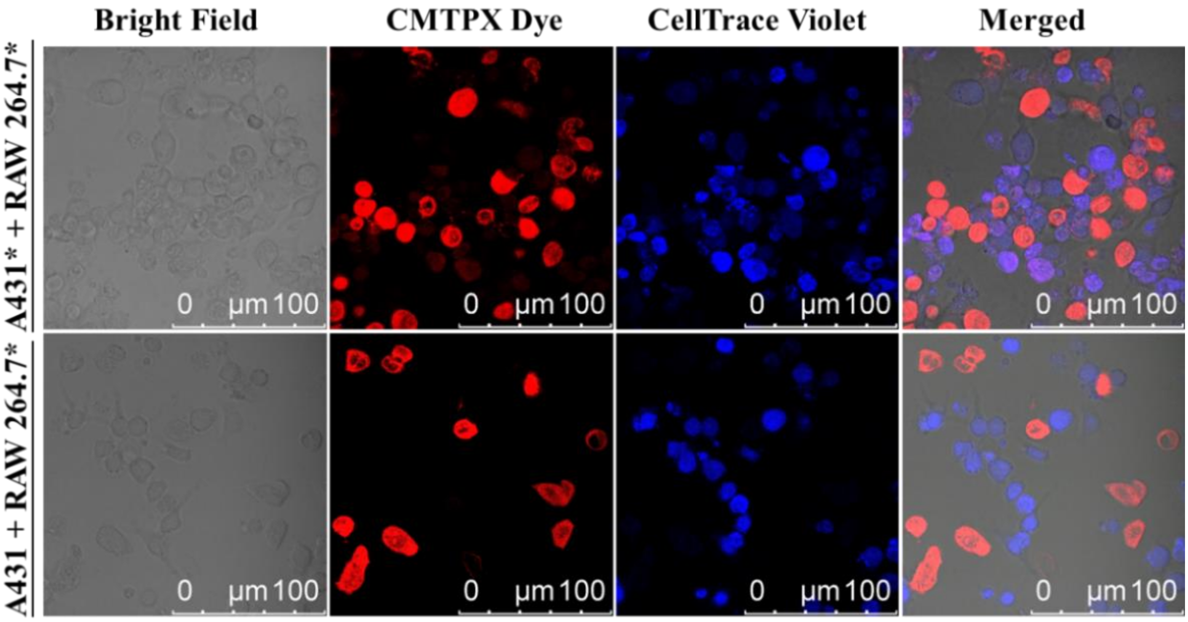

Figure S10. Confocal images of the cell assemblies of A431* (treated with 40 μM of **Mal-BCN**) or the unmodified A431 cells and LPS-treated RAW 264.7* cells co-cultured in 1:1 ratio for 30 min, followed by incubation in the culture medium for 12 h. The A431 and RAW 264.7 cells were stained with CellTracker Red CMTPX Dye and CellTrace Violet to give red and blue fluorescence, respectively.

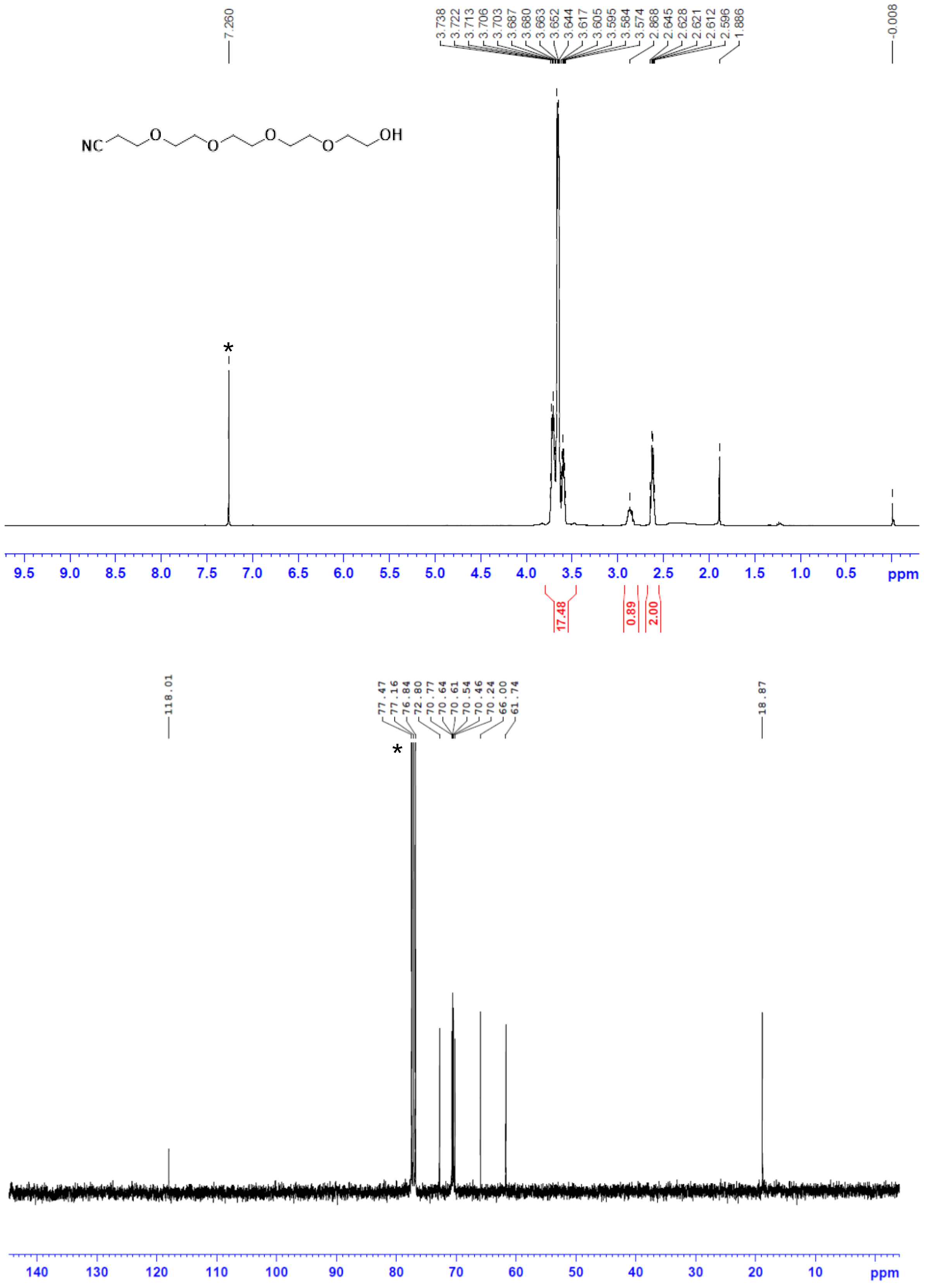

Figure S11. ^{1}H and ^{13}C{^{1}H} NMR spectra of **7** in $CDCl_3$.

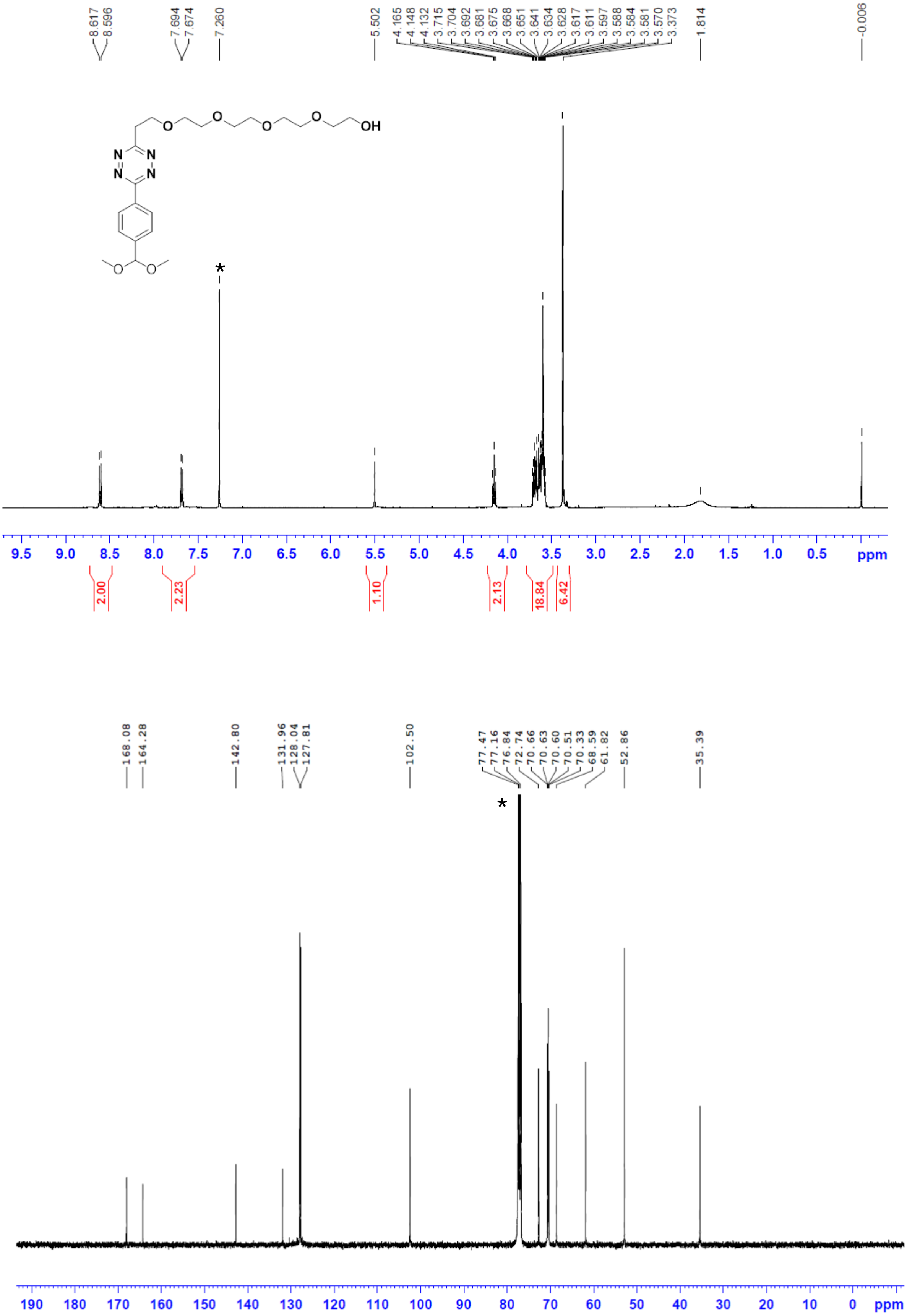

Figure S12. ^{1}H and $^{13}C\{^1H\}$ NMR spectra of **9** in $CDCl_3$.

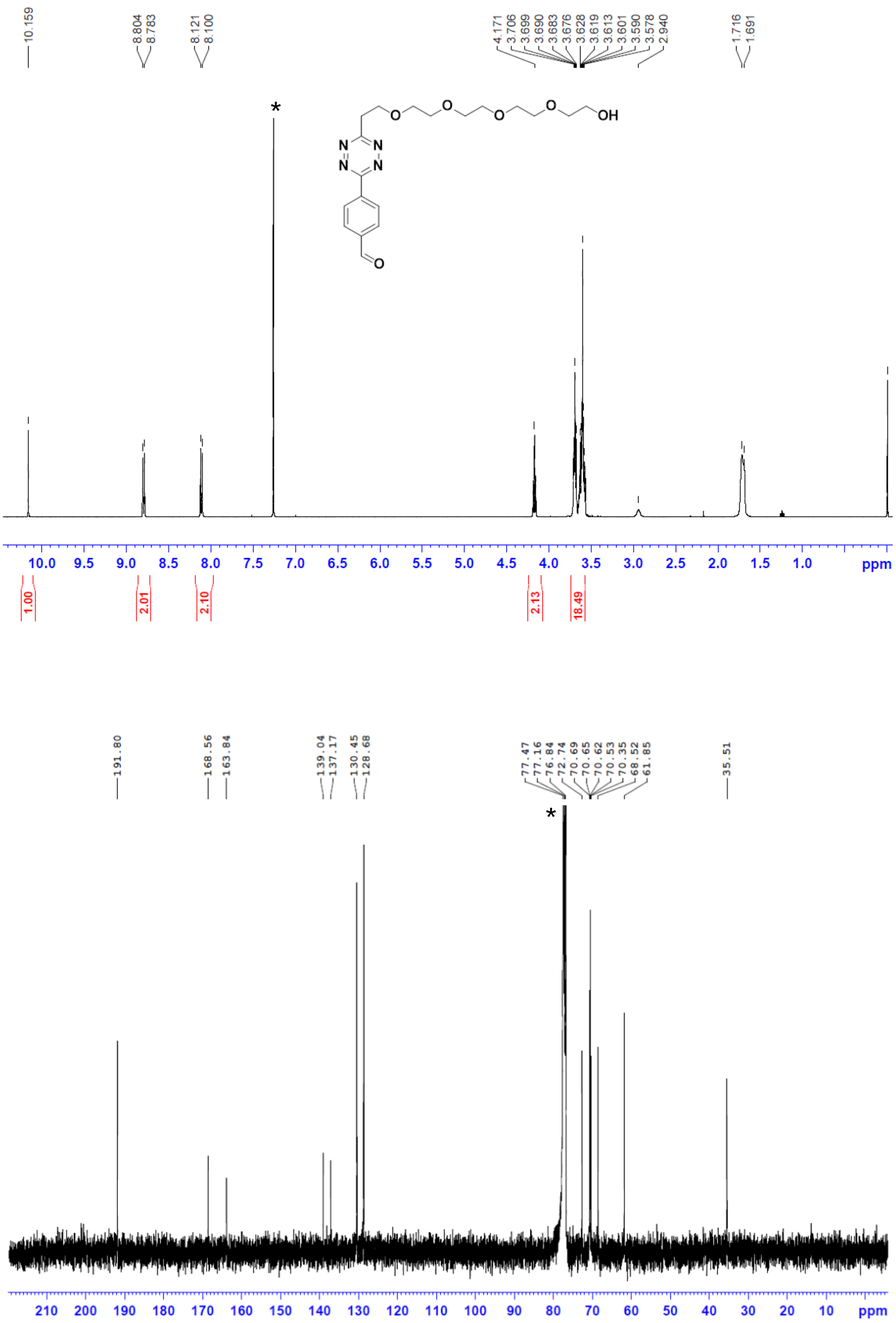

Figure S13. ^{1}H and $^{13}C\{^{1}H\}$ NMR spectra of **10** in $CDCl_3$.

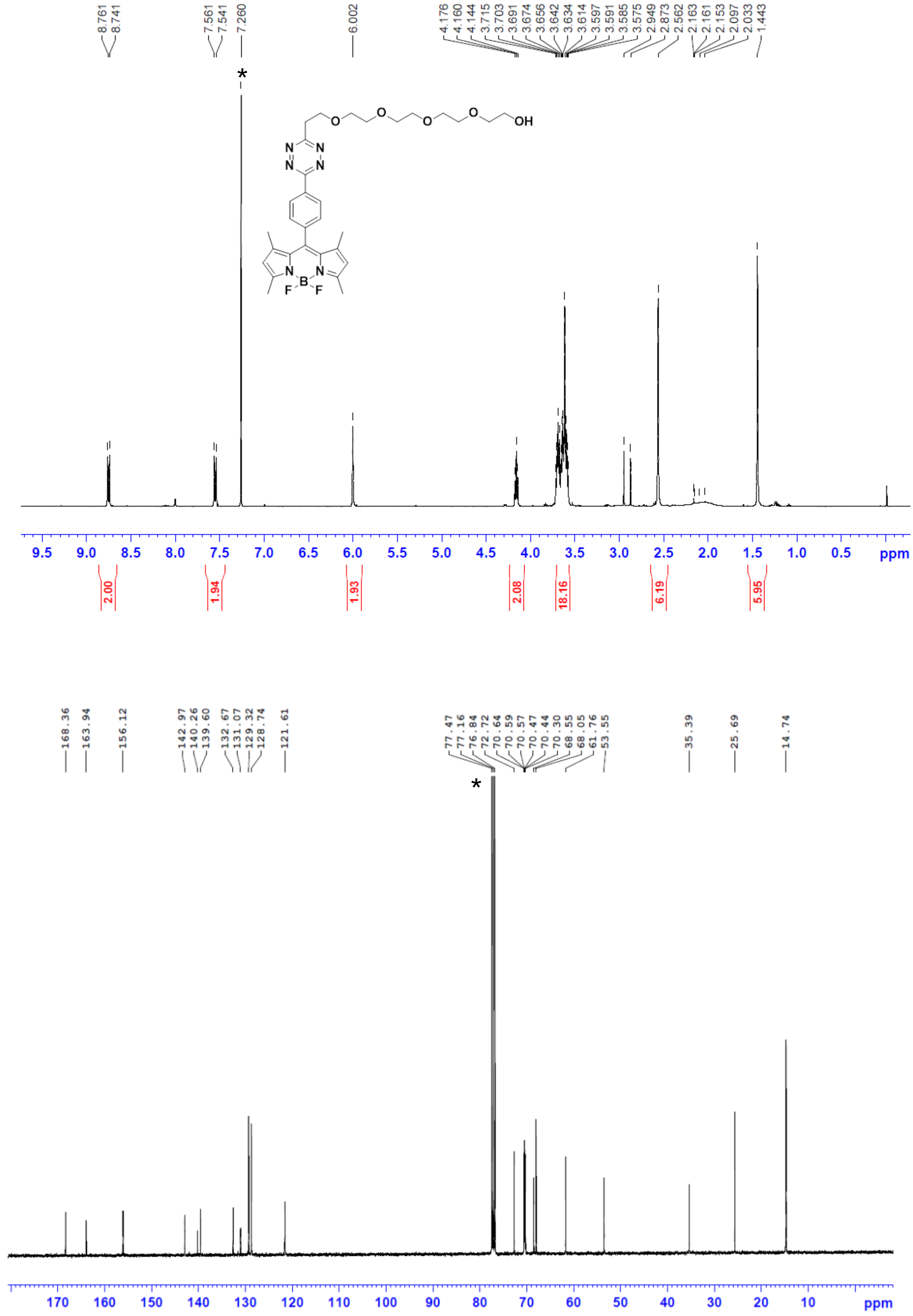

Figure S14. ^{1}H and $^{13}C\{^{1}H\}$ NMR spectra of **12** in $CDCl_3$.

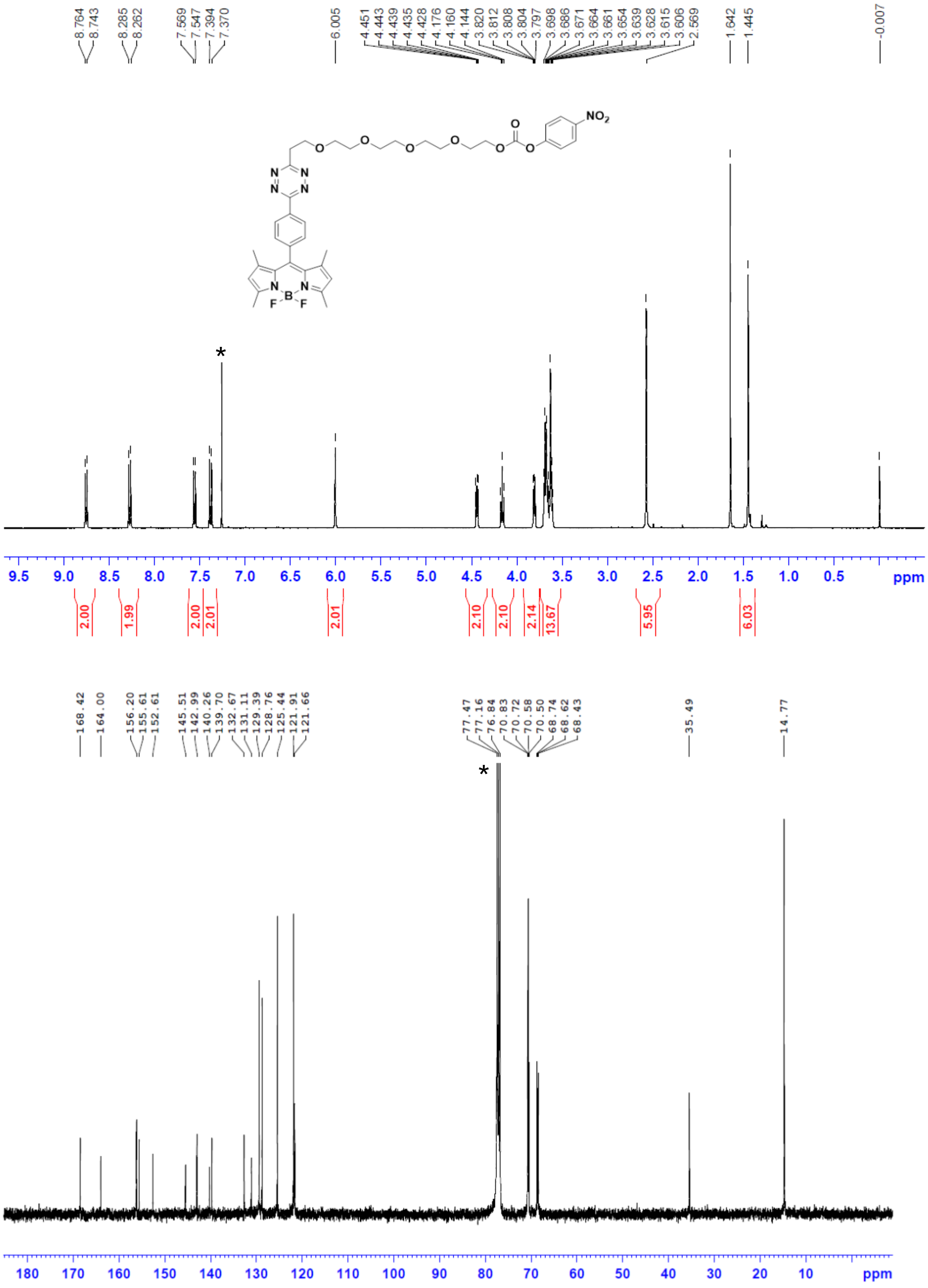

Figure S15. ^{1}H and $^{13}C\{^{1}H\}$ NMR spectra of **14** in $CDCl_3$.

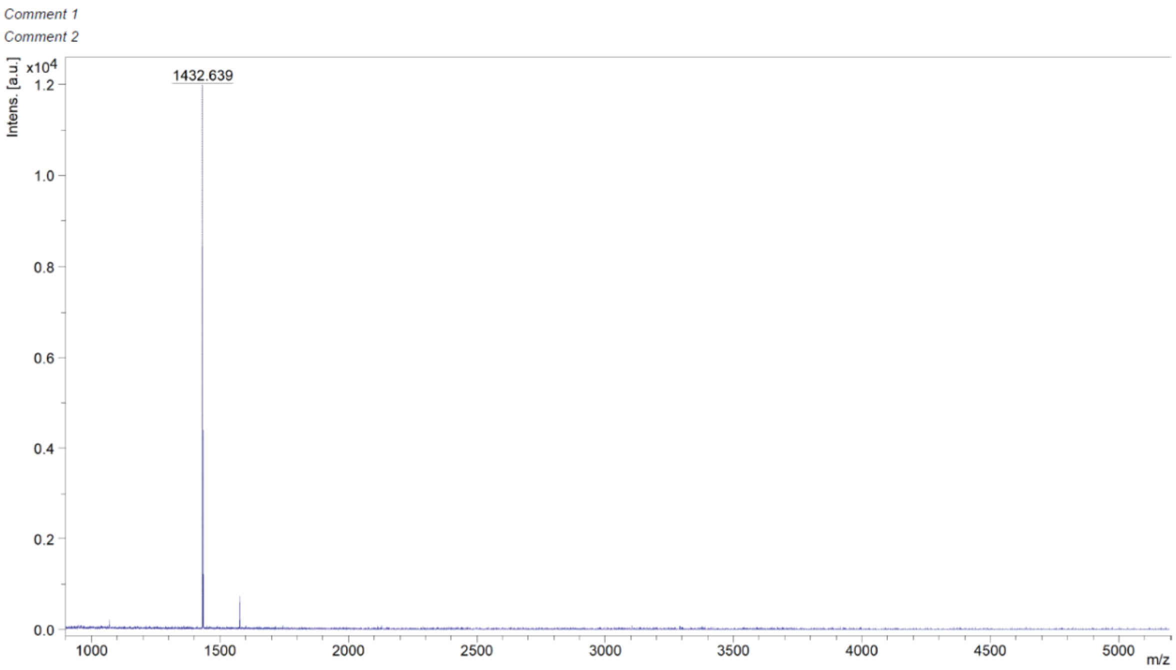

Figure S16. MALDI-TOF mass spectrum of **3**.

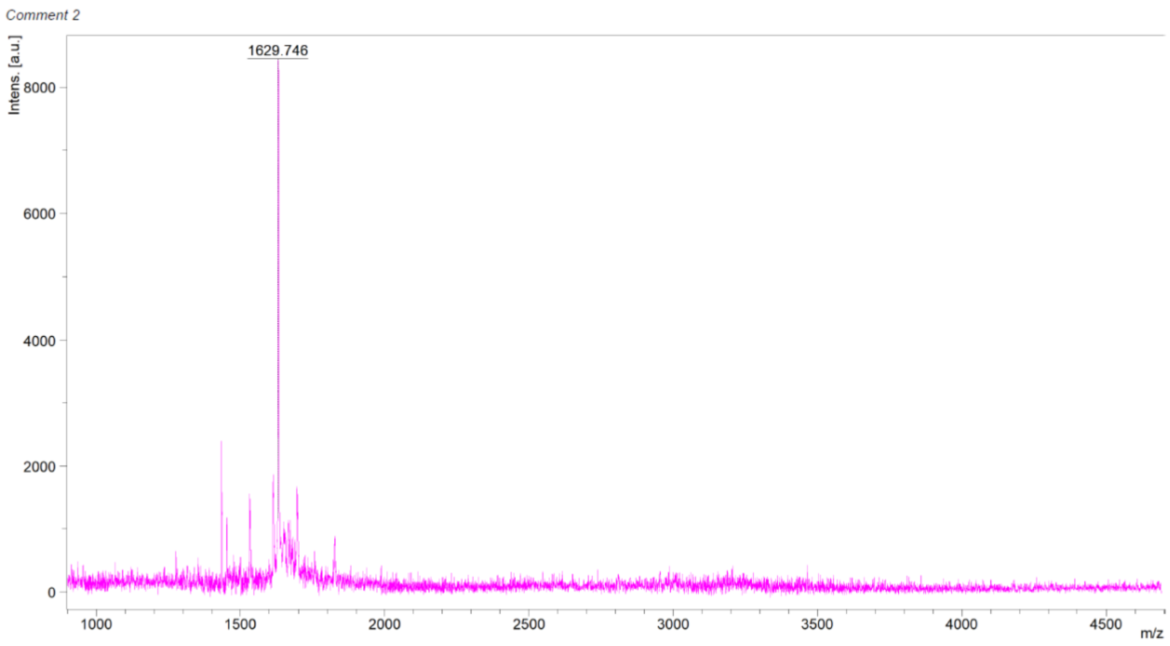

Figure S17. MALDI-TOF mass spectrum of **Mal-BCN**.

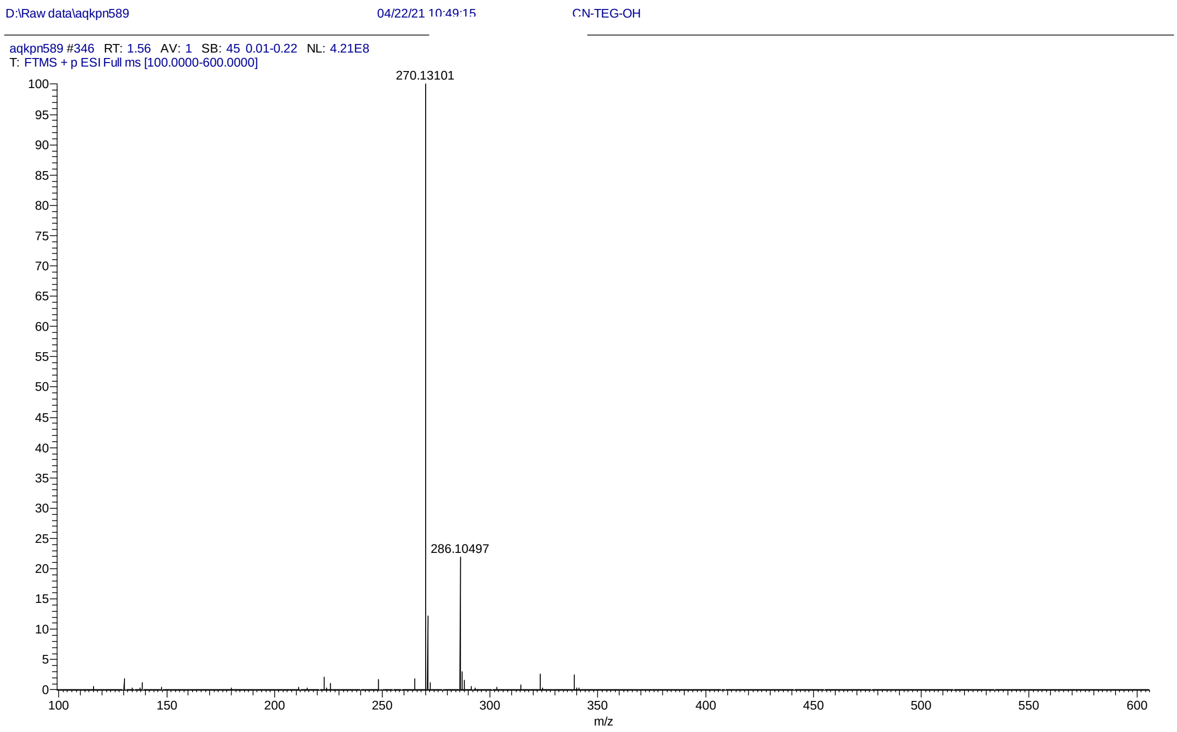

Figure S18. ESI mass spectrum of **7**.

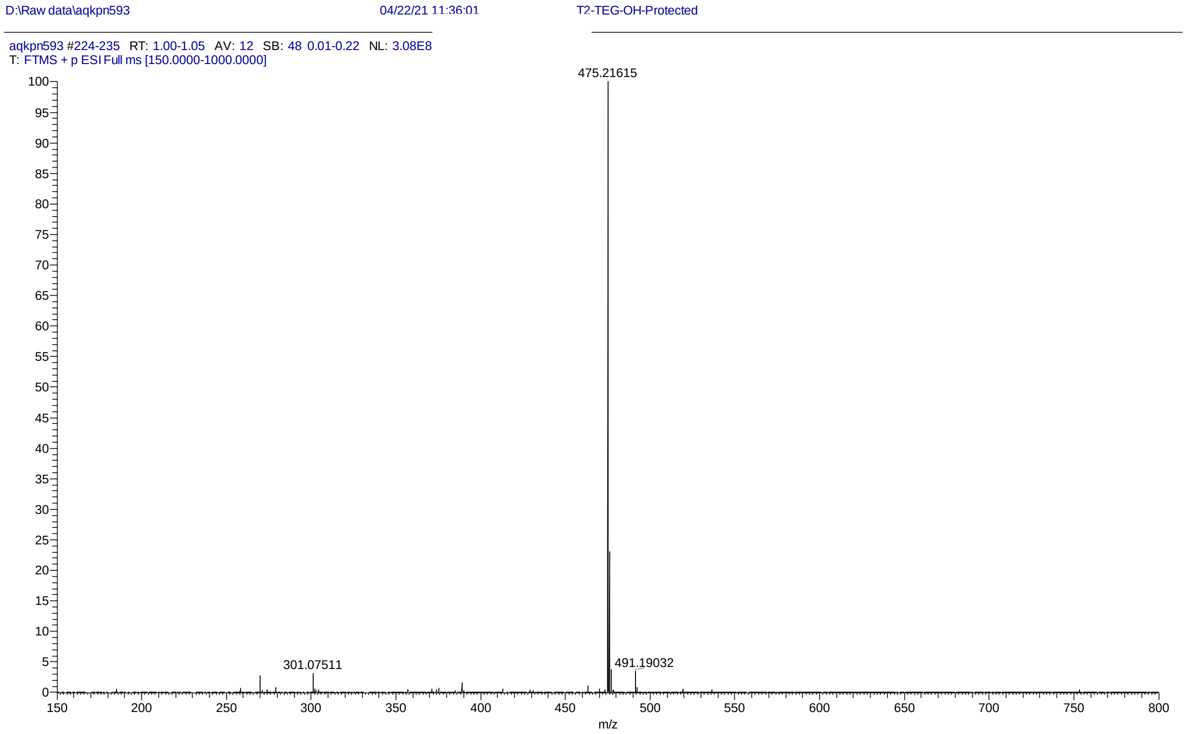

Figure S19. ESI mass spectrum of **9**.

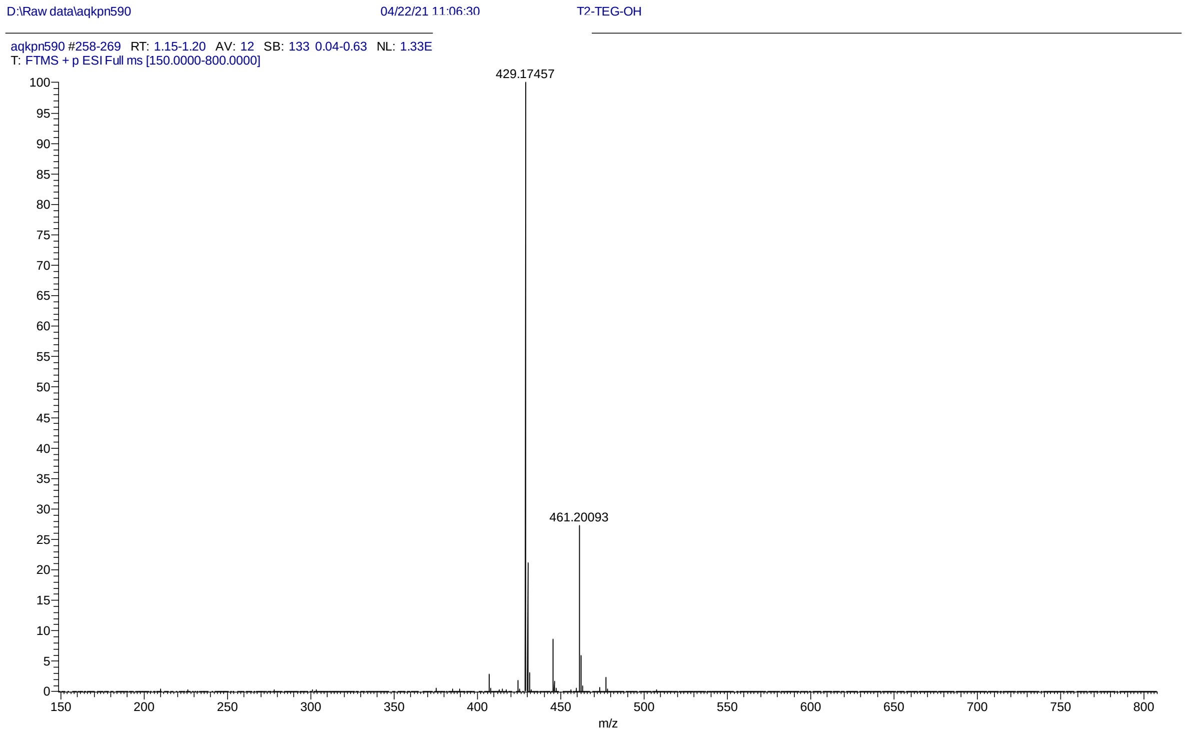

Figure S20. ESI mass spectrum of **10**.

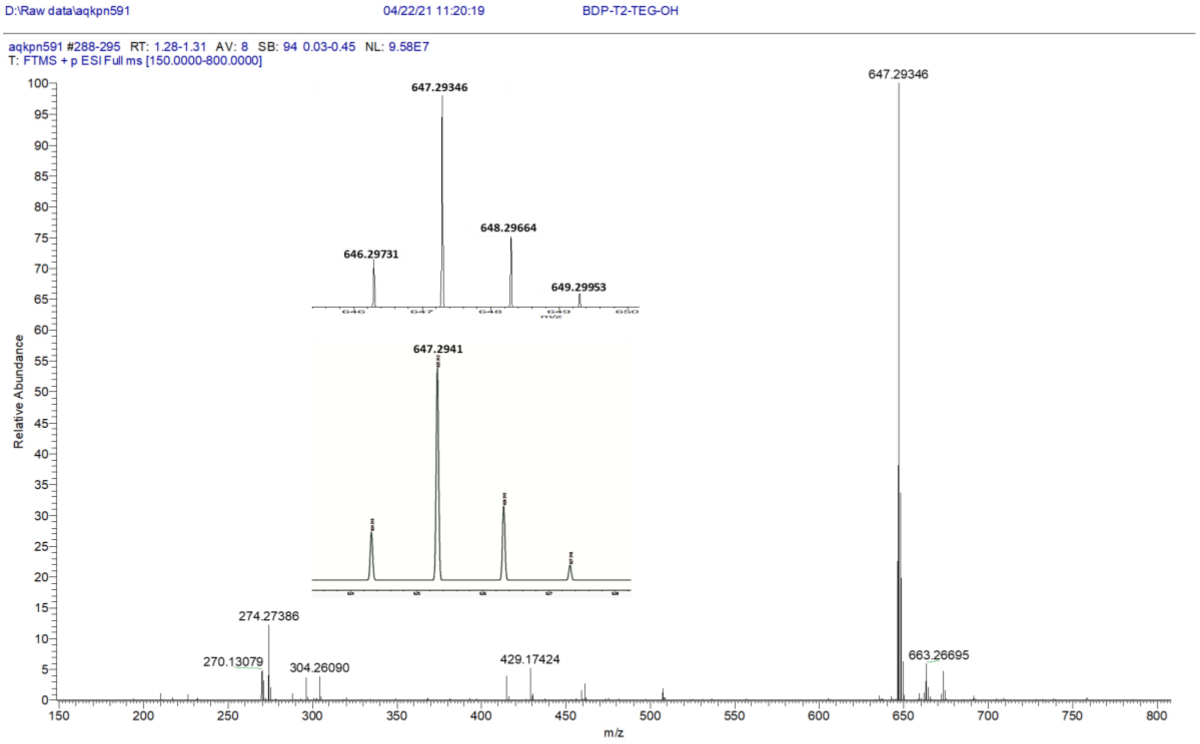

Figure S21. ESI mass spectrum of **12**. The inset shows the experimental (bottom) and calculated (top) isotopic patterns of the sodiated molecular ion $[M+Na]^+$.

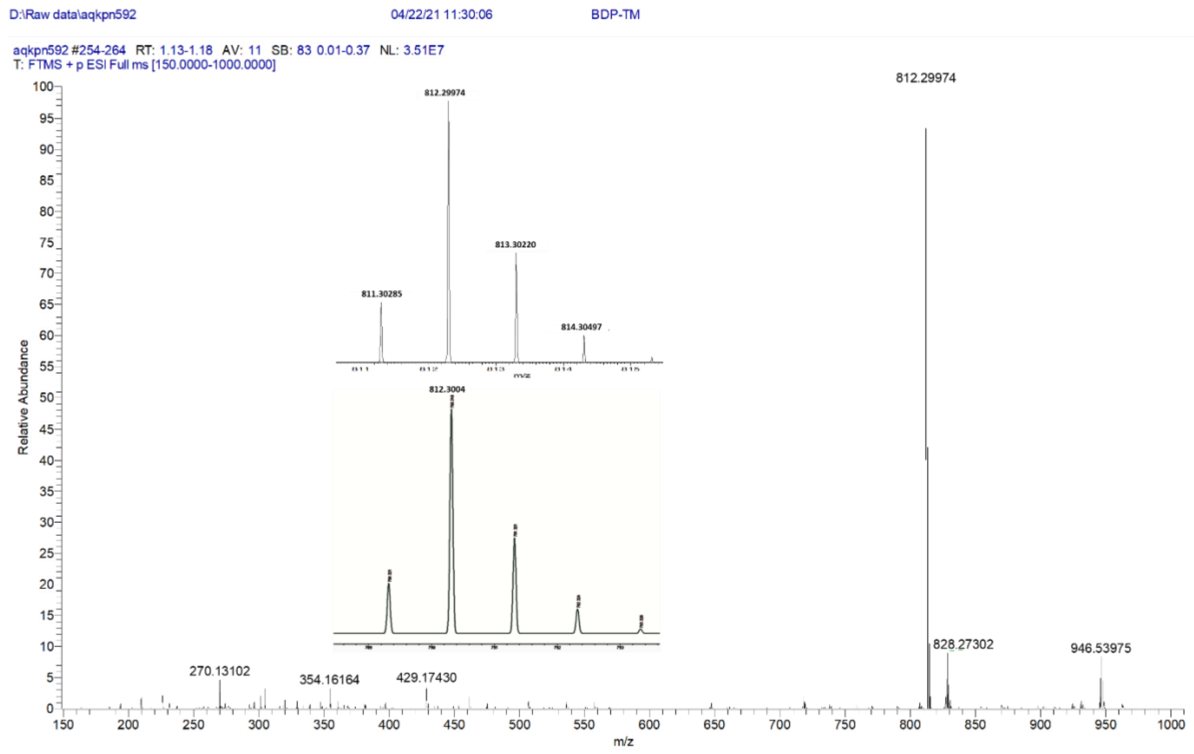

Figure S22. ESI mass spectrum of **14**. The inset shows the experimental (bottom) and calculated (top) isotopic patterns of the sodiated molecular ion $[M+Na]^+$.

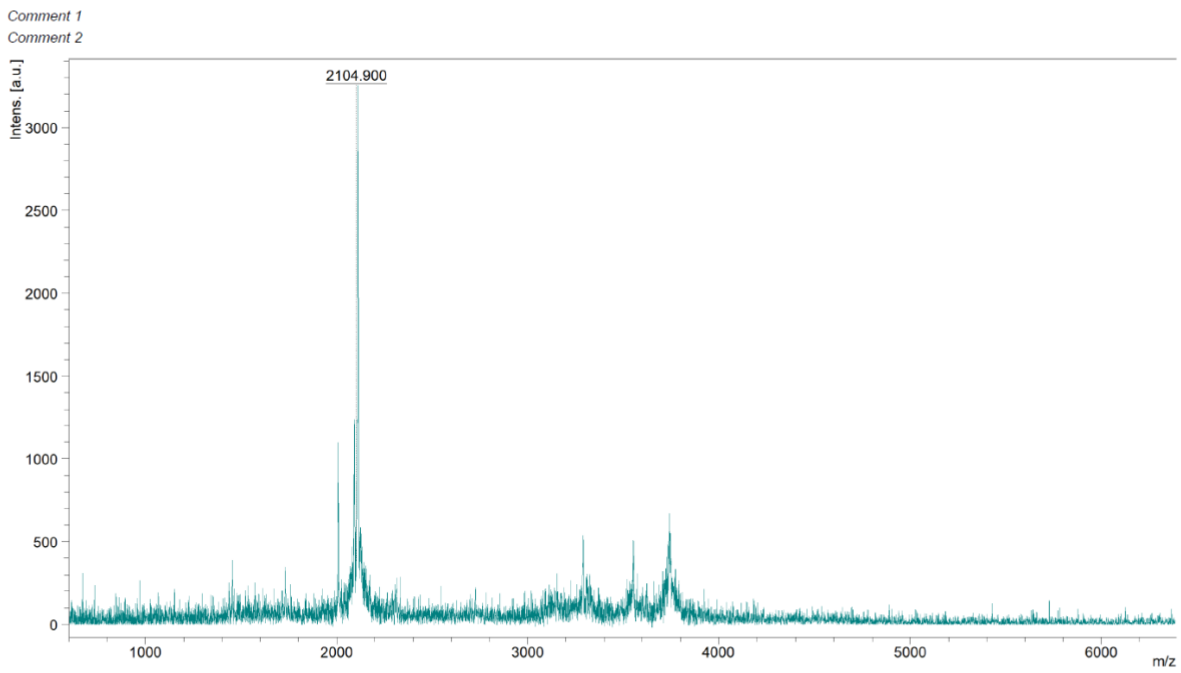

Figure S23. MALDI-TOF mass spectrum of **Mal-TzBDP**.

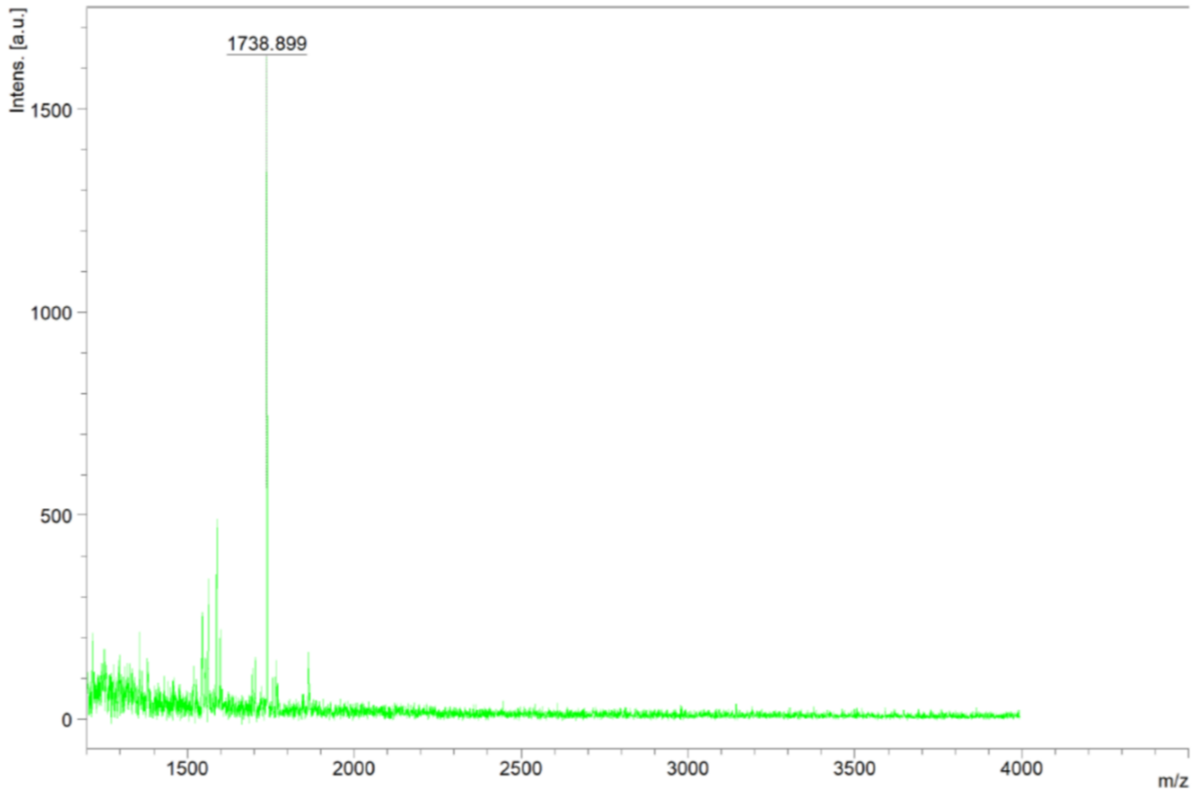

Figure S24. MALDI-TOF mass spectrum of **GE11-BCN**.

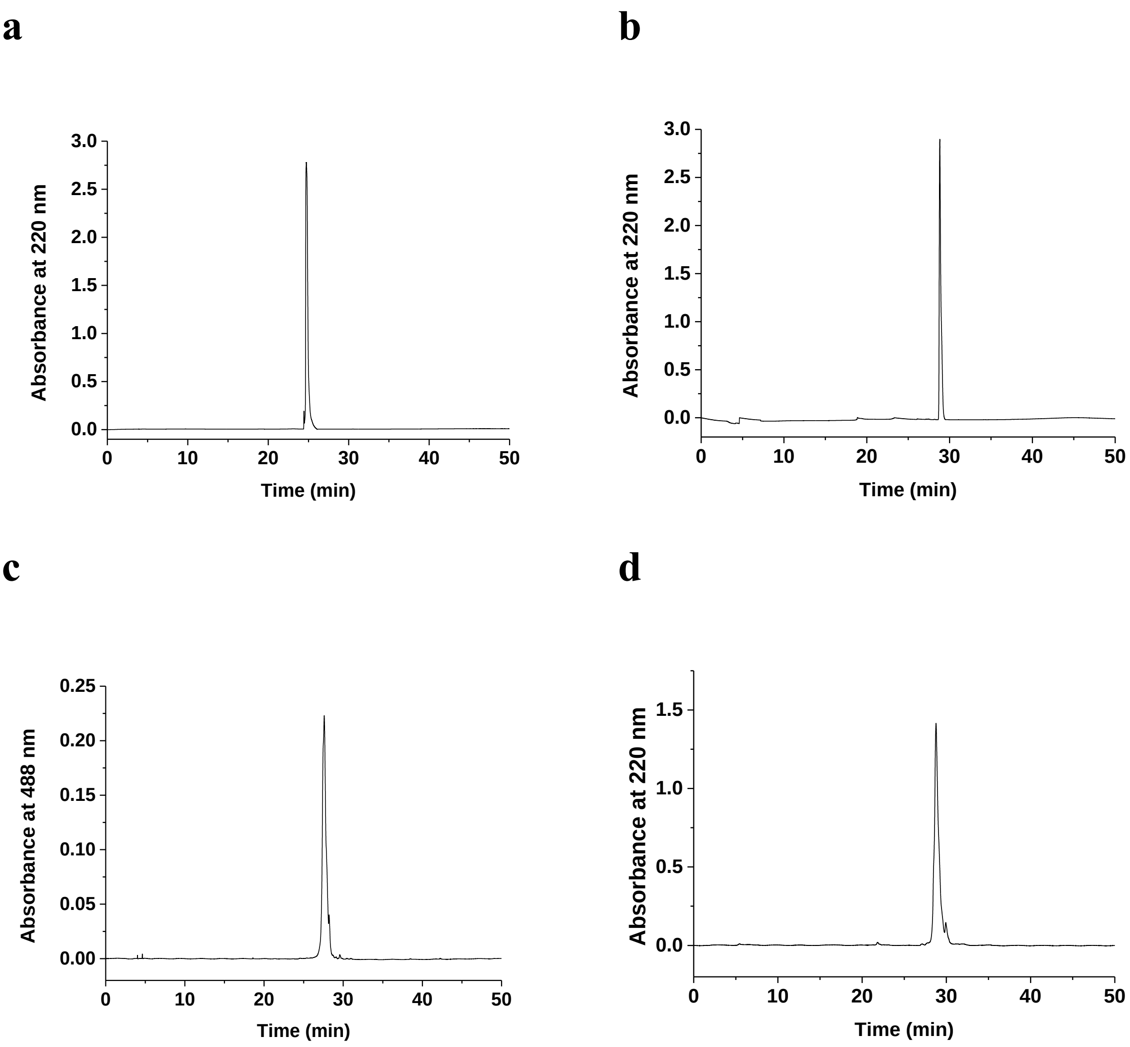

Figure S25. HPLC chromatograms of (a) **3**, (b) **Mal-BCN**, (c) **Mal-TzBDP**, and (d) **GE11-BCN.**
